# Surgical appropriateness nudges: Developing behavioral science nudges to integrate appropriateness criteria into the decision making of spine surgeons

**DOI:** 10.1371/journal.pone.0300475

**Published:** 2024-04-19

**Authors:** Teryl K. Nuckols, Peggy G. Chen, Kanaka D. Shetty, Harsimran S. Brara, Neel Anand, Nabeel Qureshi, David L. Skaggs, Jason N. Doctor, Joshua M. Pevnick, Anne F. Mannion, Surgical Appropriateness Nudge Authorship Group

**Affiliations:** 1 RAND Corporation, Santa Monica, CA, United States of America; 2 Cedars-Sinai Medical Center, Los Angeles, CA, United States of America; 3 Kaiser Permanente, Los Angeles Medical Center, Los Angeles, CA, United States of America; 4 Leonard D. Schaeffer Center for Health Policy & Economics, University of Southern California, Los Angeles, Los Angeles, CA, United States of America; 5 Schulthess Klinik, Zürich, Switzerland; National Technical University of Athens: Ethniko Metsobio Polytechneio, GREECE

## Abstract

**Background:**

Substantial variation exists in surgeon decision making. In response, multiple specialty societies have established criteria for the appropriate use of spine surgery. Yet few strategies exist to facilitate routine use of appropriateness criteria by surgeons. Behavioral science nudges are increasingly used to enhance decision making by clinicians. We sought to design “surgical appropriateness nudges” to support routine use of appropriateness criteria for degenerative lumbar scoliosis and spondylolisthesis.

**Methods:**

The work reflected Stage I of the NIH Stage Model for Behavioral Intervention Development and involved an iterative, multi-method approach, emphasizing qualitative methods. Study sites included two large referral centers for spine surgery. We recruited spine surgeons from both sites for two rounds of focus groups. To produce preliminary nudge prototypes, we examined sources of variation in surgeon decision making (Focus Group 1) and synthesized existing knowledge of appropriateness criteria, behavioral science nudge frameworks, electronic tools, and the surgical workflow. We refined nudge prototypes via feedback from content experts, site leaders, and spine surgeons (Focus Group 2). Concurrently, we collected data on surgical practices and outcomes at study sites. We pilot tested the refined nudge prototypes among spine surgeons, and surveyed them about nudge applicability, acceptability, and feasibility (scale 1–5, 5 = strongly agree).

**Results:**

Fifteen surgeons participated in focus groups, giving substantive input and feedback on nudge design. Refined nudge prototypes included: individualized surgeon score cards (frameworks: descriptive social norms/peer comparison/feedback), online calculators embedded in the EHR (decision aid/mapping), a multispecialty case conference (injunctive norms/social influence), and a preoperative check (reminders/ salience of information/ accountable justification). Two nudges (score cards, preop checks) incorporated data on surgeon practices and outcomes. Six surgeons pilot tested the refined nudges, and five completed the survey (83%). The overall mean score was 4.0 (standard deviation [SD] 0.5), with scores of 3.9 (SD 0.5) for applicability, 4.1 (SD 0.5) for acceptability, and 4.0 (SD 0.5), for feasibility. Conferences had the highest scores 4.3 (SD 0.6) and calculators the lowest 3.9 (SD 0.4).

**Conclusions:**

Behavioral science nudges might be a promising strategy for facilitating incorporation of appropriateness criteria into the surgical workflow of spine surgeons. Future stages in intervention development will test whether these surgical appropriateness nudges can be implemented in practice and influence surgical decision making.

## Introduction

Substantial variation exists in surgeon decision making regarding which patients may be good candidates for spine surgery and which spine operations may be best for each patient. For example, orthopedic surgeons and neurosurgeons, when provided with case scenarios for degenerative spinal conditions, varied greatly in when to offer surgery and which procedures to recommend [1–5]. Clinical practice patterns align with these survey findings: sizeable geographic variations exist in rates of common operations, including for lumbar spine procedures, and are not explained by differences in patient populations [2, 6–8]. Across hospital referral regions, rates of elective lumbar spinal decompression and fusion have varied by 8.6- and 14-fold, respectively, among Medicare beneficiaries [7] Among people who underwent laminectomy for lumbar spondylolisthesis, rates of concomitant fusion have ranged from 82% to 98% across states. Operative outcomes and costs also exhibit substantial variation [9]. Use of complex lumbar spinal fusion procedures, instead of less complex ones, has been linked to higher rates of complications [10].

Created in response to unexplained variation in surgical care, appropriateness criteria are tools that provide recommendations about the balance of benefits and risks of a specific procedure for an individual patient [11, 12]. Appropriateness is an aspect of the quality of health care. Rigorously developed appropriateness criteria have high validity, reliability, and a long history of use in research. Across many conditions, adherence to such criteria has been associated with improved operative outcomes [11, 13–17]. Spine surgery specialty societies have made appropriateness criteria and supporting tools publicly available for diverse procedures [18, 19]. Yet, as with guidelines, many barriers hinder surgeons from using appropriateness criteria in routine practice [20].

To overcome such barriers, behavioral science “nudges” have emerged as a promising strategy for enhancing clinical decision making [21]. Behavioral science strives to understand how people make decisions and act on them, and how to overcome common shortfalls in decision making. A nudge is defined as a modest adjustment to the environment that influences behavior in a predictable way without limiting autonomy [22]. Literature has classified diverse nudges according to “frameworks” that reflect how the nudges shape behavior as well as the anticipated strength of different types of nudges. Examples of nudge frameworks include drawing attention to social norms, providing default settings, giving reminders, and providing feedback [21, 23–30]. Widely used in other settings, evidence has grown that nudges can be effective tools for shaping clinician behavior including promoting adherence to guidelines and other recommended standards of care [21–35].

This project sought to conduct exploratory work to generate and begin to refine “surgical appropriateness nudges.” These represent a novel intervention that would leverage behavioral science nudges to support spine surgeons’ routine use of appropriateness criteria for degenerative lumbar scoliosis and spondylolisthesis and thereby shape the surgeons’ decision-making and behavior [36, 37]. If nudges are eventually found to be effective at increasing the appropriateness of surgical procedures, operative outcomes may improve.

## Materials and methods

The National Institutes of Health (NIH) Stage Model for Behavioral Intervention Development proposes best practices for generating, testing, and implementing interventions that are effective at shaping human behavior in real-world settings. Stage I includes generating and refining an intervention, while later stages involve initial experimental tests that maximize internal validity, larger experimental tests in community settings that maximize external validity, and finally research on strategies for promoting adoption of the now evidence-based intervention [38, 39]. The current work corresponded to Stage I. Characteristically, the earliest phase of behavioral intervention development involves an iterative, multi-method approach that leverages literature reviews, taxonomies of intervention elements, input from content experts, qualitative methods such as focus groups drawn from populations that would receive the intervention, and data on current behaviors and outcomes, among other potential inputs and resources. Hallmarks of this stage include allowing the intervention to remain fluid, to permit ongoing refinements in response to evolving findings, and performing initial tests with a small number of highly selected participants [38, 40].

Consistent with this stage, qualitative methods were the primary methods used in this research. Qualitative methods enable researchers to explore behaviors and interactions surrounding complex topics in depth [41, 42], particularly when the full range of potential responses is not known *a priori* [43]. Because the proposed application of behavioral science nudges had not been well studied, it was important to remain open to opportunities, challenges, and facilitators to nudge implementation in a surgical setting. Specifically, we conducted focus groups because they are ideal for the initial exploratory phases of intervention design and allow participants’ opinions to evolve over the course of discussions with peers [41, 42].

Our work adhered to widely accepted standards for rigor in qualitative research [41, 42, 44], and the Standards for Reporting Qualitative Research (SRQR) guidelines [45]. To recruit participants, site leaders invited 89 orthopedists and neurosurgeons who performed procedures for degenerative lumbar scoliosis and/or spondylolisthesis. We sought 5–7 respondents per site to allow sufficient opportunities to engage each surgeon while maximizing diversity in specialty, career stage, gender, and race/ethnicity [46]. To facilitate the focus groups, we developed discussion guides with questions and probes to elicit debate. Questions were open-ended, allowing latitude in exact wording, sequencing of questions, and use of probes while ensuring important domains were consistently addressed [47].

We conducted focus groups on Microsoft Teams and each group lasted approximately 60 minutes. All focus groups were audio-recorded and professionally transcribed to ensure accuracy and fidelity to the original discussion. For this study, because we sought to ensure our understanding of specific processes and insights on specific nudges, we focused on ensuring content saturation within each focus group discussion. We continued each line of questioning until participants had no additional input and pro-actively solicited input from each participant before progressing to the next topic. We utilized commercially available software (Dedoose) to manage coding, retrieval and analysis. In accordance with principles of grounded theory [48], experts in qualitative methods (co-authors PC and NQ) developed a code structure in stages using systematic, inductive procedures to generate insights grounded in participants’ views. We coded the first transcript independently and met to discuss differences in coding, making edits to the code structure as needed to reach consensus. We then divided the remaining transcripts, meeting regularly to discuss any coding challenges and identify emergent and recurrent themes. We utilized the constant comparative approach to identify novel concepts [48], consistently classify emergent themes, and refine or expand existing codes as needed. We began with a review of discussion notes highlighting potential early themes and maintained a running list of themes, making edits and consolidating and refining themes when appropriate.

Institutional Review Boards at the RAND Corporation and the two study sites approved this work. One study site required written informed consent while the other site allowed for verbal consent.

### Setting

We partnered with two high-volume regional referral centers for spine surgery that have a strong commitment to the quality and outcomes of surgical care and where leaders sought to implement appropriateness criteria in routine practice. At both sites, neurosurgeons and orthopedists care for complex degenerative spine disorders. Site 1 receives referrals from throughout southern California, Nevada, and Arizona. Site 2 is a capitated, integrated healthcare system with 4.5 million members. Both sites use Epic electronic health record systems (EHRs).

### Nudge design process

We sought to develop nudges that spine surgeons would view as applicable to their clinical practice, feasible for incorporation in the surgical workflow, and acceptable to the surgeons personally for routine use.

**Fig 1:**
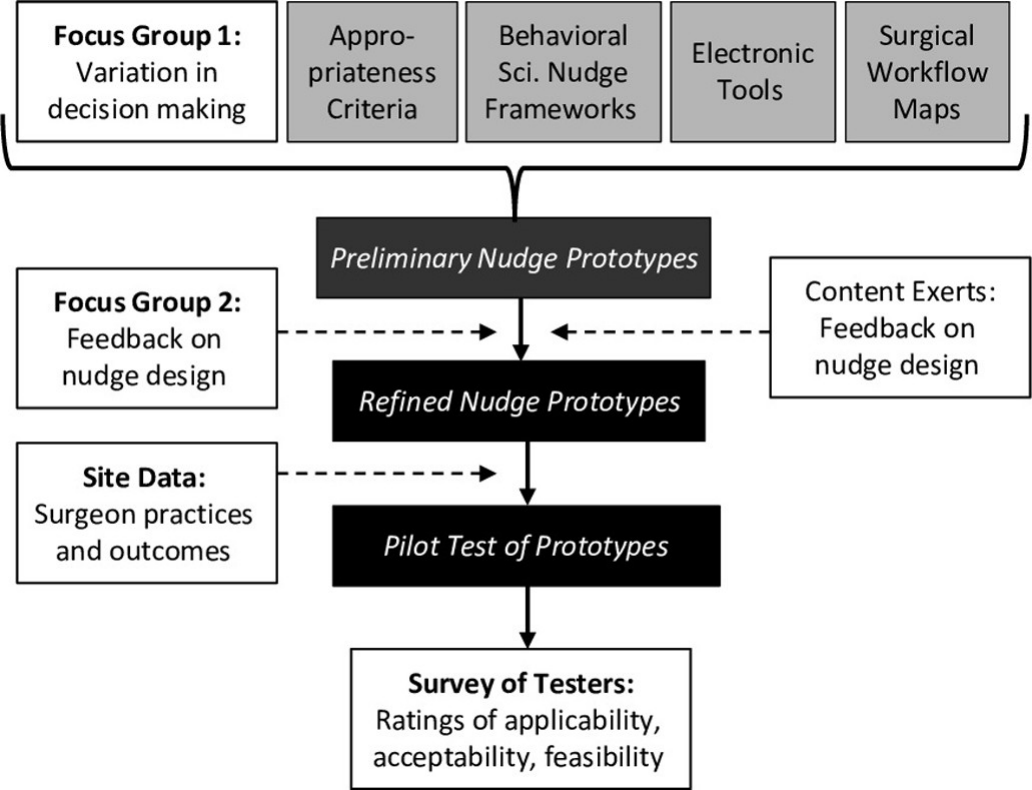
Flow of information in the design of surgical appropriateness nudges.

As seen in **Fig 1**, the first step included conducting Focus Group 1 and synthesizing existing knowledge. Focus Group 1 involved asking spine surgeons to describe the surgical workflow and identify sources of variation in surgeon decision making because these could represent opportunities to insert nudges to enhance decision making. We considered four kinds of existing knowledge: (1) appropriateness criteria for degenerative lumbar scoliosis and spondylolisthesis [37, 49], (2) frameworks for behavioral science nudges, (3) electronic tools that can facilitate clinical decision making, and (4) maps of the surgical workflow at study sites.

In the second step of nudge design, we synthesized focus group results and existing knowledge to propose preliminary nudge prototypes.

The third step involved soliciting feedback on preliminary nudge prototypes from the content experts and site leaders as well as from spine surgeons via Focus Group 2. We incorporated the feedback to produce several refined prototypes.

Concurrent to the intervention development work, we gathered data on spine surgeon practices and outcomes at the study sites.

In the fourth step of nudge development, we pilot tested the refined nudge prototypes among several spine surgeons at each site. Some nudge prototypes incorporated the data on surgeons’ practices and outcomes. Finally, we surveyed the testers to assess perceptions of the prototypes and obtained additional feedback. We elaborate upon each step below.

To maximize the likelihood that we considered all potentially effective nudge designs and that insights obtained could translate to other settings, we solicited feedback from multiple external experts throughout the nudge design process. This included an Advisory Board of national experts and stakeholders in surgical quality of care, representatives of national specialty societies engaged in relevant work on appropriateness of spine surgery, and collaborators in Switzerland, among others.

### Focus Group 1: Surgeon decision making

In focus group 1, we sought a rich understanding of surgeons’ beliefs on decision making about appropriateness, including sources of variation as well as implications for practice patterns and patient outcomes. We also presented draft workflow maps and asked participants to make edits and suggestions to better understand key decision making steps in the surgical workflow.

### Synthesis of existing knowledge

#### Appropriateness criteria

In general, these criteria classify the balance of benefits and risks of a specific procedure for 100-1000s of “indications profiles” (scenarios) based on patient characteristics (e.g., history, physical exam, test results), thereby precisely defining the treatment options that are safe to offer an individual patient. The criteria provide recommendations about whether, for a specific indications profile, a particular procedure is “appropriate” (benefits exceed risks), “rarely appropriate” (risks exceed benefits, sometimes called “inappropriate”), or of “uncertain appropriateness” (risks/benefits are uncertain or mixed).

In prior stages of this work, our team and the Schulthess Klinik in Switzerland (led by co-author AM) independently used the well-established RAND/UCLA Appropriateness Method to develop criteria for degenerative lumbar scoliosis and spondylolisthesis, respectively [36, 37, 49, 50]. This method synthesizes guidelines, published literature, and expert opinion [11, 12, 15, 16, 51–55]. The scoliosis criteria address five procedures (decompression, fusion, fusion + decompression, fusion + deformity correction, and fusion + deformity correction + decompression) in 260 scenarios based on seven characteristics (symptoms, severity and extent of stenosis, progression, sagittal imbalance, risk factors, and curvature) [37, 56]. The spondylolisthesis criteria address 372 scenarios based on nine characteristics (similar to scoliosis) and yield recommendations for three procedures (decompression, fusion +/-decompression, and instrumented fusion +/- decompression) [36, 49]. The American Academy of Orthopaedic Surgeons (AAOS) formally endorsed the scoliosis criteria in 2016 [57].

In the present work, we leveraged these two sets of appropriateness criteria. We sought feedback from study spine surgeons about validity and applicability of the criteria (during pilot tests of nudge prototypes, see below). Additionally, we searched for publications on adherence to these appropriateness criteria and other relevant appropriateness criteria.

#### Behavioral science nudge frameworks

We conducted a purposive literature review to identify key studies on nudges, frameworks (types of nudges), applications in healthcare, and pros/cons of specific nudges. Sources on behavioral science and nudges in general included widely referenced books and articles [21, 22, 58–63]. For applications in healthcare, we searched PubMed using the terms “behavioral economic*”, “behavioral science”, and “nudg*” as title words (March 18, 2022). Because the results from this search appeared to capture most recent literature applicable to healthcare, we did not conduct an independent systematic review. A content expert in behavioral science (co-author JD) provided input at key stages.

#### Electronic tools to support clinician decision making

We considered two potential platforms for supporting use of nudges: online calculators and tools based in the EHR.

**Fig 2:**
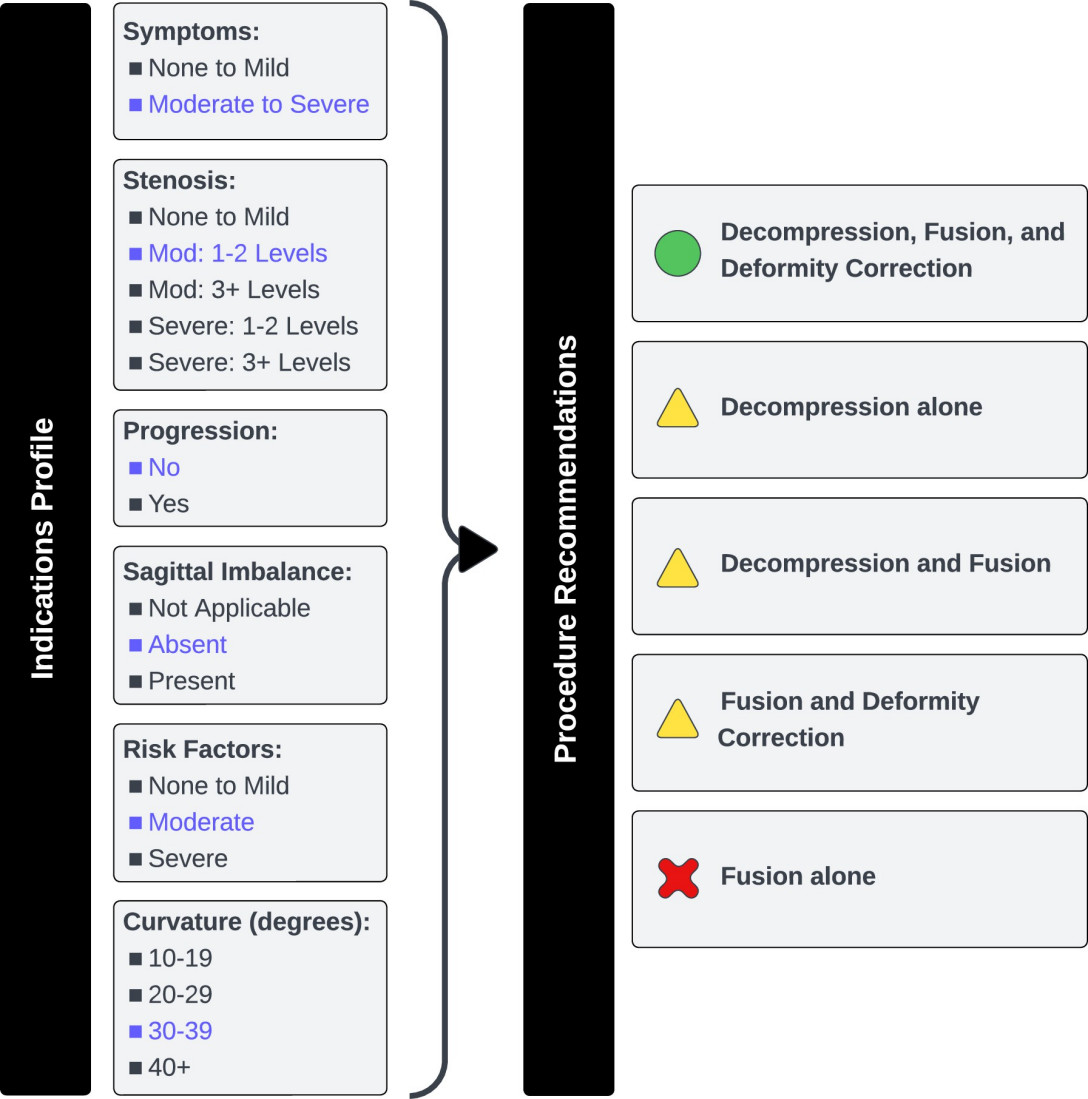
Online appropriateness calculator for degenerative lumbar scoliosis. ***Indications Profile*:** The user enters specific clinical characteristics for an individual patient. These include symptoms, age, comorbidities, physical exam signs, imaging test results, and other factors that influence the risks and benefits of a specific procedure for that person. The blue font reflects a profile for a hypothetical patient. ***Procedure Recommendations*:** The calculator uses the indications profile to score the appropriateness criteria and yields recommendations for common surgical options. For the hypothetical patient, a green circle means that the procedure is “appropriate” (potential benefits exceed risks), the yellow triangle means that a procedure is of “uncertain appropriateness” (risk-benefit ratio is unclear or mixed), the red X means that the procedure is “rarely appropriate” (risks exceed potential benefits). Note: The American Academy of Orthopaedic Surgeons produced online calculators based on the appropriateness criteria for degenerative lumbar scoliosis and spondylolisthesis. This graphic explains how the appropriateness criteria and associated calculators work. The graphic itself is original artwork by study investigators.

In prior stages of this work, the American Academy of Orthopaedic Surgeons (AAOS) created online appropriateness calculators for the scoliosis and spondylolisthesis appropriateness criteria (**Fig 2**) [19, 64].

To understand how the appropriateness criteria or online calculators could be delivered to clinicians via the EHR, we conferred with a content expert experienced with Epic systems (co-author JP).

#### Surgical workflow maps

We created draft maps of workflows at both sites by interviewing site leaders (co-authors HB, NA), identifying key decisions and their timing, and creating a graphic. We refined the maps based on input from spine surgeons via Focus Group 1 (above).

### Preliminary nudge prototypes

After Focus Group 1, we designed preliminary nudge prototypes in several iterative steps. We reviewed lists of nudge frameworks, prior applications of nudges in healthcare, surgical workflows, sources of variation in surgical practice, and prior deployments of nudges via the EHR. We identified opportunities for nudges to influence practice and to leverage existing or novel tools. We considered evidence on the effectiveness of diverse nudges at shaping clinician behavior and how that might apply to surgeons and complex decisions. We used the PreDICT Checklist, a tool designed to help ensure that surgeons’ implicit or explicit preferences/concerns are addressed in designing the nudge [63]. We considered nudges more likely to be acceptable if transparent and convenient to use. We excluded nudges that were poorly suited to complex decision-making processes.

With input from the surgeon site leaders and content experts, we integrated the assembled information, added any newly suggested nudge frameworks or prototypes, considered the pros/cons of each nudge framework, and up-/down-prioritized them based on major pros/cons. Because interventions that involve multiple nudges are often effective [25], we selected several frameworks, suggested prototypes of how they could be operationalized in the surgical workflow, and created tables and graphics.

### Focus Group 2: Feedback from spine surgeons

Using the same participants and methods as for Focus Group 1, we presented participants with background information on behavioral science and nudge frameworks that had been effective in other healthcare settings. Next, we shared the preliminary nudge prototypes. Surgeons provided substantive, constructive feedback on nudge design, nudges’ potential acceptability, feasibility, and effectiveness; their preferences among nudges; and potential refinements.

### Refined nudge prototypes

We incorporated the surgeons’ feedback and made additional iterative modifications based on assembled information and input from site leaders and content experts.

The three nudges that emerged from Focus Group 2 were not considered strong, according to the framework ranking nudge strength [21], since surgeons would have to actively choose to engage each nudge and to change their behavior. Additionally, none of the nudges would be delivered to surgeons in real time during decision making for individual patients. Given these limitations, we conferred with site leaders and content experts to add a stronger and more active type of nudge that we had not offered to focus group participants.

### Surgical practices and outcomes at study sites

In parallel to the nudge design work, we examined surgeon-level variations in practices and outcomes for degenerative lumbar scoliosis and spondylolisthesis at study sites for 2017–2019. In work reported separately [65], we extracted ICD-10-CM and CPT codes from EHR systems, characterized surgeon-level variations in practice (surgeon volume for study conditions, proportion of procedures involving instrumented fusion) and short-term postoperative outcomes (major in-hospital complications, readmissions).

We also collected preliminary data on adherence to appropriateness criteria for 12 spine surgeons divided equally between study sites: half participated in the pilot tests of nudge protypes (below) and half were selected at random. For each surgeon, we randomly selected 5 patients (60 total) from the 2017–2019 dataset. We created a self-correcting data collection instrument in Microsoft Excel and then trained clinicians to manually apply the instrument to medical records in the EHR.

### Initial pilot testing of refined prototypes

Among the surgeons who participated in the focus groups, we sought six volunteers (three per site) to pilot test four refined nudge prototypes, tailoring the testing approach for each nudge.

One prototype involved the AAOS online calculators. We asked each surgeon to use the calculators with ≥5 clinic patients for whom they were considering surgery (≥30 patients total). In feedback meetings with the study team, the surgeons shared their experiences, commenting on both the appropriateness recommendations as well as usability of the calculators.

Two prototypes involved providing the participating surgeons with individualized feedback about their practices and outcomes relative to peers at study sites, and their adherence to appropriateness criteria for individual patients. First, we designed the documents to deliver the feedback. Next, we incorporated the 2017–2019 data from study sites (above) into the documents, and then provided the participants with the individualized feedback.

One nudge involved a conference on appropriateness. Because surgeons routinely participate in similar conferences, we provided the participating surgeons with a detailed one-page description of how the appropriateness conference would function.

After the pilot test activities, we surveyed the six participating surgeons about the refined nudge prototypes. We adapted an existing instrument that is widely used to assess implementation outcomes [66]. For each of the refined nudge prototypes, our survey included eight items related to nudge applicability, feasibility, and acceptability, scored on a 1–5 scale (5 = strongly agree). We distributed the survey via REDCap. We also solicited qualitative feedback about testers’ experiences.

## Results

Fifteen surgeons voluntarily participated in the focus groups across the two sites. Surgeons were diverse with regards to age, gender and specialty (e.g., neurosurgery and orthopedic surgery). Six of these surgeons voluntarily participated in the pilot tests.

### Focus Group 1: Surgeon decision making

Surgeons believed that institutional protocols and procedures, the availability of specialists, and reimbursement models affected surgical decision making. The main surgeon characteristic highlighted was experience, with more senior surgeons reporting that they became more conservative and relied more on clinical symptoms and signs rather than imaging. Patient factors included patients’ priorities, out-of-pocket costs, and age/comorbidities. See **S1 File** for detailed results of Focus Group 1.

### Synthesis of existing knowledge

#### Appropriateness criteria

Surgeons who participated in the pilot tests did not report concerns about the validity or applicability of the criteria.

We identified two publications that had employed the study appropriateness criteria: the Schulthess Klinik and a Dutch group found that 18–40% of operations were inappropriate, and that appropriateness was associated with better patient-reported outcomes [67, 68]. In earlier studies, 14–49% of lumbar spine operations were found to be inappropriate [14, 54, 68].

#### Behavioral science nudge frameworks

The purposive search yielded nine recent systematic reviews [21, 23–30], five protocols or conceptual analyses, and other literature [69–74]. The nudge frameworks were similar across publications, with modest differences. One resource ranked the “strength” of different frameworks, meaning their likelihood of influencing behavior [21].

#### Electronic tools to support clinician decision making

Surgeons who pilot tested the AAOS’ online calculators suggested several modest improvements in formatting.

We identified three Epic-based platforms that could deliver nudges. A “smart phrase” can facilitate consistent documentation, bring in data from elsewhere in the EHR, and include embedded links (nudge framework: defaults). A best practice alert is a type of interruptive clinical decision support (nudge framework: reminders/alerts). The final platform was putting a link to online appropriateness calculators in an easy-to-find EHR menu (nudge framework: decision aid/mapping).

## Surgical workflow maps: See S2 File

### Preliminary nudge prototypes

Our iterative process produced five preliminary prototypes that incorporated several nudge frameworks: (1) *individualized score cards (descriptive norm/peer comparison/feedback)* that report surgeon’s practices and outcomes relative to peers; (2) *online appropriateness calculators* with links embedded in the EHR *(decision aid/mapping)*, (3) *structured note templates (defaults)* that include prompt surgeons to document the clinical variables needed to assess appropriateness; (4) *prompts to document a rationale for any exceptions (accountable justification)* if a planned operation was not aligned with appropriateness criteria; (5) *multispecialty case conference (injunctive norm/social influence)* to discuss exemplar cases, appropriateness criteria, and recent publications. We considered and excluded additional nudge frameworks because they were poorly suited to highly complex decision making.

#### Focus Group 2: Feedback from spine surgeons

Surgeons provided in-depth feedback and narrowed the list of five preliminary nudge prototypes to three that appeared most promising: embedding links to the online calculators in the EHR, individualized surgeon score cards, and multispecialty case conferences (**S3 File** includes detailed results of Focus Group 2).

*Individualized score cards*. This nudge generated much discussion among participants—surgeons felt that score cards should be clear and transparent in their development, argued that they should not be publicly reported, and were most interested in comparing themselves with close peers.

*Online appropriateness calculators*. Surgeons generally viewed these favorably. A barrier to use was that surgeons would need to remember to access the calculators, sometimes from diverse computer terminals.

*Structured note template*. Surgeons had mixed reactions, reporting that documentation practices varied greatly. Several surgeons did not write visit notes themselves, but rather asked spine fellows and physician assistants to do so, and variable documentation practices would hamper implementation.

*Rationale for exceptions*. Focus group participants did not have strong reactions for or against this, but no promising opportunity to incorporate this in the workflow had been identified.

*Multispecialty case conference*. Surgeons were receptive, based on their experiences with case conferences from residency/fellowship training. However, they doubted that they often performed “rarely appropriate” procedures or omitted highly “appropriate” ones. But they did often wrestle with the many situations where appropriateness was uncertain and wanted these to be discussed in case conferences too.

### Refined nudge prototypes

**Fig 3:**
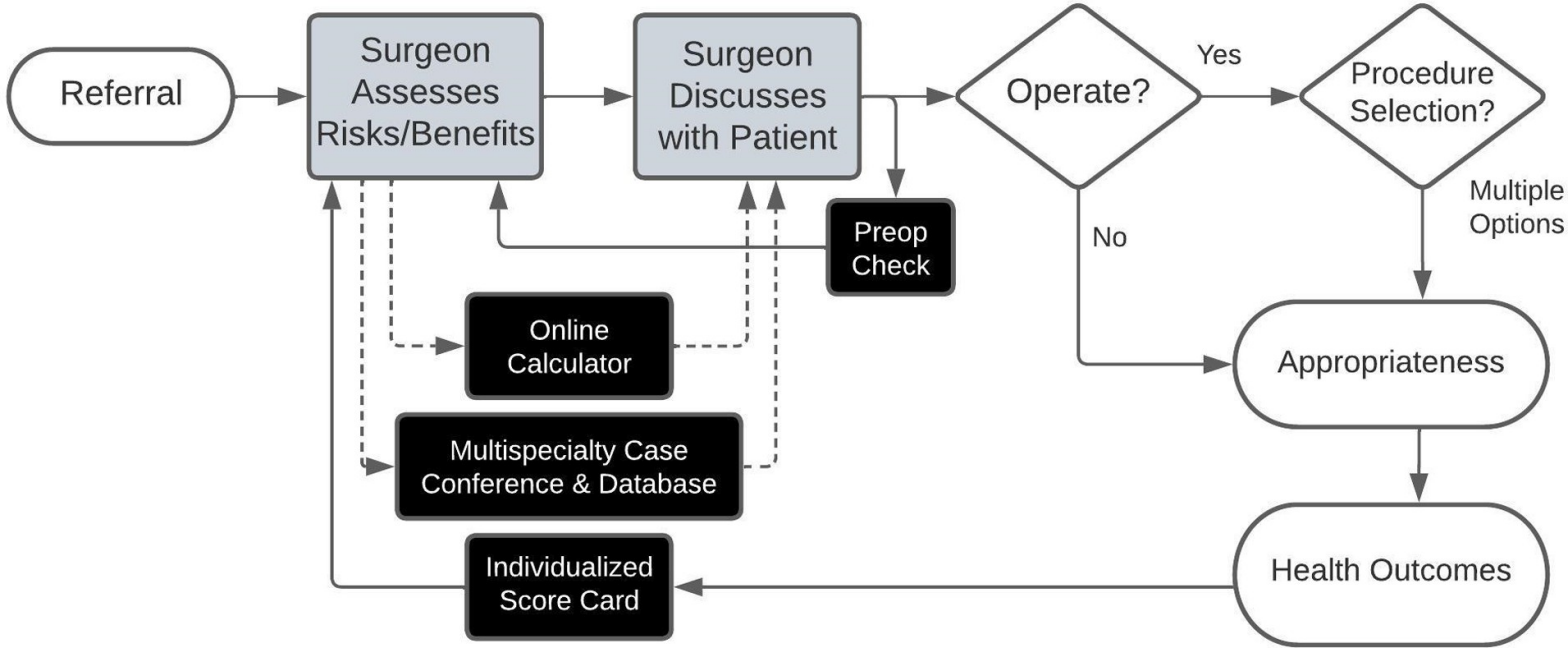
Refined surgical appropriateness nudges in preoperative workflow. ***Workflow*:** After being referred a patient with degenerative lumbar scoliosis and/or spondylolisthesis (white oval), the surgeon assesses the risks and benefits of performing surgery and of alternative surgical procedures (grey rectangle). Based on this assessment, the surgeon discusses treatment options with the patient (grey rectangle). Based on this discussion, the surgeon then operates or not (white diamond) and, if operating, selects among multiple procedure options (white diamond). The surgical care provided may or may not align with appropriateness criteria recommendations (white oval). The appropriateness of surgical care influences the patient’s health outcomes (white oval). ***Nudges*:** Individualized Score Cards and the Multispecialty Case Conferences (black rectangles) can inform the surgeon’s overall approach to assessing risks and benefits. Surgeons can access Online Calculators and Multispecialty Case Conference Database during the assessment of risks/benefits and selection of procedure for an individual patient. The Preop Check delivers recommendations to the surgeon shortly after the procedure has been scheduled.

Based on Focus Group 2, three nudges emerged most promising: online calculators embedded in the EHR, individualized surgeon score cards, and multispecialty case conferences. Because these nudges are of only weak to moderate strength, we later added a fourth, stronger nudge that we had not presented to the focus group participants: a preoperative (“preop”) check. **Table 1** summarizes the refined nudge prototypes, while **Fig 3** shows how they fit into the spine surgery workflow and ultimately shape surgical practice and outcomes. The **S4 File** includes a table summarizing the nudge frameworks considered in this work, those included vs. excluded, and a rationale for each decision. Additionally, the **S5 File** includes details about the refined nudge prototypes including examples.

**Table 1:** Refined prototypes of surgical appropriateness nudges: Design and characteristics.

Design	Characteristics
***Individualized Score Cards*:** These would show each surgeon’s adherence to appropriateness criteria as well as practices (use of instrumented fusion) and outcomes (major in-hospital complications) relative to peers, framed to draw the surgeon’s attention. Score cards would be updated regularly, delivered privately, and mask the identities of peers. Reviewing score card data may encourage surgeons to respond to other nudges.	**Nudge Frameworks:** Descriptive norm†/ Peer comparison/ Feedback **Flexibility/Strength:** * Provided to all surgeons for optional review; moderate strength **Timing:** Delivered to surgeons at baseline and quarterly
***Online Appropriateness Calculator Embedded in Electronic Health Record System*:** These would be embedded in the EHR via links in an easy-to-find menu. Surgeons would enter clinical variables in the calculator, which would then score the appropriateness criteria and display the appropriateness of 3–5 alternative procedures. Surgeons could elect to use the resulting procedure recommendations to educate patients about appropriate treatment options.	**Nudge Frameworks:** Decision aid/ Mapping **Flexibility/Strength:** Provided to all surgeons for optional use; weaker **Timing:** Offered to surgeons for use during visits before surgery, with continuous access via links in the EHR
***Multispecialty Case Conference and Database*:** Members would include diverse specialties with substantial experience in both operative and non-operative approaches to treating degenerative spine conditions: orthopedic-trained spine surgeons, neurosurgery-trained spine surgeons, physiatrists, pain management experts, geriatricians, primary care physicians, psychiatrists/psychologists, and possibly physical therapists. Via online teleconference, the committee would select and discuss exemplar cases reflecting diverse scenarios. The appropriateness criteria and updated literature searches would inform deliberations. A searchable database would enable surgeons to access the archived case, with case characteristics, appropriateness criteria classification and recommendations, recent literature, and deliberations. Surgeons would also be able to self-refer cases for review.	**Nudge Frameworks:** Injunctive norm‡/ Social influence **Flexibility/Strength:** All surgeons are invited to attend conferences, can consult the database as desired, and can self-refer cases for review; weaker **Timing:** Surgeons invited to monthly conferences; offered continuous access to the database
***Preop Check*:** Scheduling an operation would trigger the preop check. A clinician, such as a physician assistant, would review the surgeon’s notes to assess which procedure(s), if any, are preferred for this patient. The clinician would email/message this recommendation to the surgeon. The email/message will include a link to the online appropriateness calculator, prompt surgeons document a rationale if the surgical plan diverged from the appropriateness criteria, and invite them to refer cases to the multidisciplinary care conference.	**Nudge Frameworks:** Reminder/ Salience of Information and Accountable Justification **Flexibility/Strength:** Provided to all surgeons for optional review; stronger **Timing:** Delivered to surgeons shortly after an operation is scheduled and before surgery

* Based on a ranking of the strength of nudge frameworks in an article by Last et al. (2021) [21].

† Descriptive norms represent how people actually behave in practice—in this case, their performance relative to peers [75, 76].

‡ Injunctive norms reflect perceptions of what behaviors are approved or disapproved by others—in this case a multidisciplinary team of experts [75, 76].

#### Individualized score cards

In addition to leveraging the descriptive norm, peer comparison, and feedback frameworks, viewing individualized data on performance may encourage surgeons to respond to the other nudges. Although we considered "framing” the information to emphasize the negatives, other literature suggested that the score cards would need to provide feedback in a non-judgmental, constructive way to avoid triggering defensive reactions [70, 72].

#### Online appropriateness calculators

By themselves, decision aids are weak interventions since surgeons have to remember to use them. However, as part of the other three nudges, surgeons would receive reminders about the online calculators, how to find them in the EHR, and why they would be useful.

#### Multispecialty case conference

Due to the roles of social influence and norms in shaping surgical practice, a case conference might influence the appropriateness of surgery both directly and indirectly (i.e., beyond the patients presented).

#### Preop check

Implementing the preop check requires a readily available, continuously updated data stream that can detect when surgeons are considering surgery for specific patients. The operating room schedule represents such a data stream. Accordingly, the preop check is feasible late in the surgical workflow, after the surgeon has offered surgery to the patient. Nonetheless, the preop check could still guide surgeons to the procedure with the best risk-benefit ratio, prompt surgeons to document a rationale if the surgical plan diverges from the appropriateness criteria (an “accountable justification”), and/or modify their decision making for subsequent patients.

### Surgical practices and outcomes at study sites

Across the study sites in 2017–2019, 89 spine surgeons performed 2,481 eligible operations. The surgeons exhibited substantial variation in operative volume, use of instrumented fusion, and postoperative outcomes [65]. At the two sites, the median eligible operative volume was 9 (range 1–119) and 14 (range 1–115), respectively. Higher-volume individual surgeons (≥15 eligible procedures) used instrumented fusion in 0% to >90% of their operations for scoliosis and 9% to 100% of their operations for spondylolisthesis, and they had major in-hospital complications after 0% to 25% their operations for scoliosis and 0% to 14% of their operations for spondylolisthesis (reported in detail elsewhere).

Of the 60 surgical patients in whom we tested methods for scoring appropriateness criteria, 30 had sufficient information documented in the EHR to score. Surgery was discordant with the appropriateness criteria in 16 (53%): 12 (40%) had inappropriate surgery and 4 (13%) did not undergo the best procedure. These rates are similar to published literature (above).

### Initial pilot testing of refined prototypes

**Fig 4:**
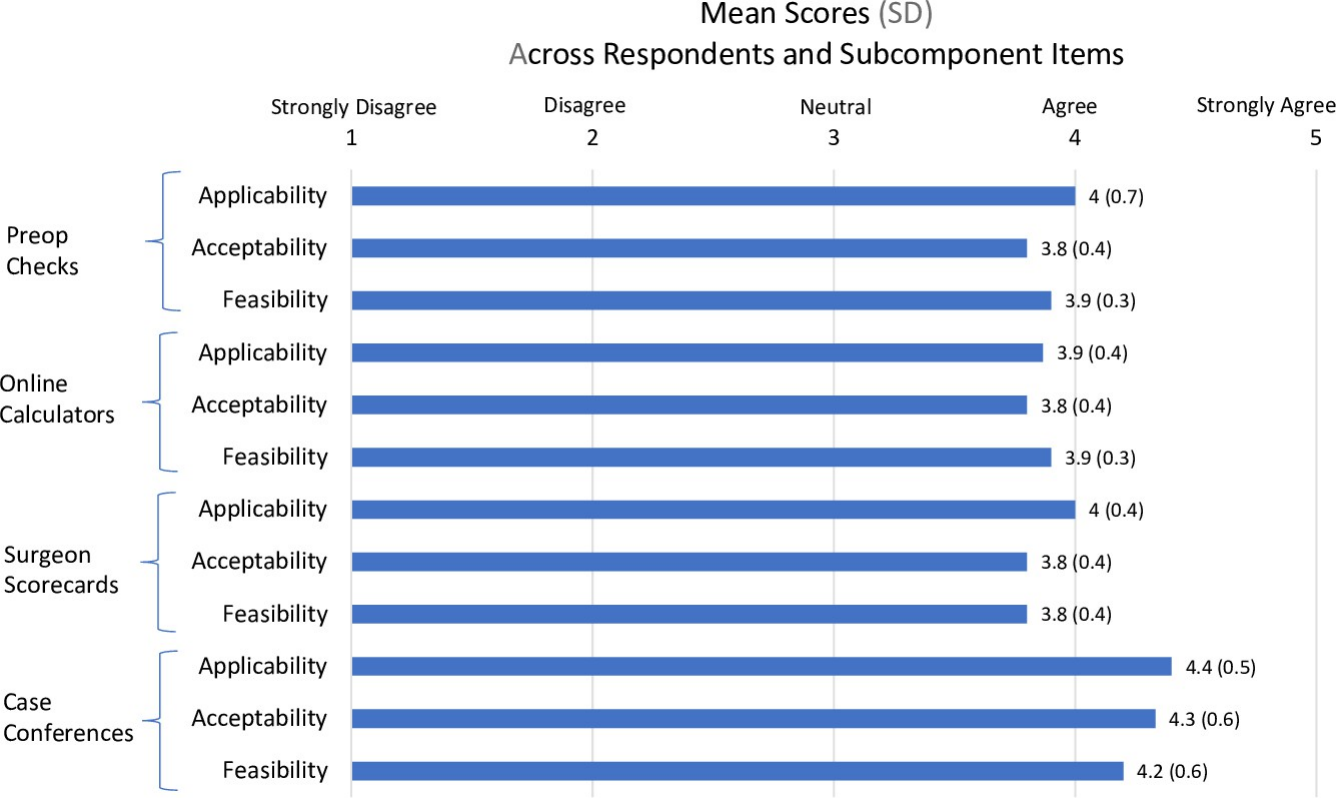
Survey of spine surgeons who pilot tested refined prototypes of surgical appropriateness nudges. For each of the four refined nudge prototypes, surgeons rated applicability (3 items), acceptability (3 items), and feasibility (2 items), using a 1–5 scale (strongly disagree to strongly agree).

Of the six surgeons who participated in pilot tests, five completed the survey (83%). Among respondents, the mean overall score was 4.0 (standard deviation [SD] 0.5), showing good overall support for the nudges (**Fig 4**). Mean scores by dimension were: applicability 3.9 (SD 0.5), feasibility 4.0 (0.5), and acceptability 4.1 (0.5). Conferences had the highest scores 4.3 (SD 0.6) and calculators had the lowest 3.9 (0.4). See **S6 File** for items and responses.

## Discussion

In this study, we designed surgical appropriateness nudges using an iterative primarily qualitative process that leveraged published literature, content experts, spine surgery leaders, and focus groups with spine surgeons at two regional referral centers, as well as data on baseline surgical practices and outcomes. Surgeons valued the ability to assess the risks and benefits of surgery for individual patients and were amenable to surgical appropriateness nudges that might help them achieve that. In particular, surgeons thought that three nudges appeared promising: individualized surgeon score cards, online appropriateness calculators, and a multispecialty case conference and database. To strengthen the set of nudges, the research team added a preop check that would give surgeons feedback about appropriateness in real time before surgery. Surgeons who pilot tested these four nudges rated them on average as applicable, acceptable, and feasible.

Diverse behavioral science nudges have been effective at shaping clinician behavior in many settings, based on nine recent systematic reviews synthesizing hundreds of studies [21, 23–30]. One review concluded that the quality of the studies was moderate overall [23], but reviews limited to Level I evidence still reported substantial effectiveness [26]. The types of nudges studied varied across reviews; the most common types included defaults, priming, social norms, reminders/salience, and providing feedback [21, 23–30].

Despite the increasing use of nudges in clinical contexts, very few studies have addressed surgical care. Studies have addressed hand hygiene in surgical ICUs, lung-protective ventilation strategies during general anesthesia, use of chlorhexidine in ventilated patients in surgical ICUs, prescribing opioids after surgery, and prescribing perioperative antibiotics [21, 23–30]. Our work appears innovative in its application of nudges to surgical appropriateness. By engaging surgeons in nudge development, we aimed to improve the reception of nudges among this group.

Sethi et al. reported a prior systematic effort to assure the appropriateness of surgery for adult scoliosis patients [77]. As one part of a multifaceted quality improvement intervention, the investigators held a case conference where clinicians from neurosurgery, anesthesia, orthopedics, internal medicine, behavioral health, and nursing met to collaboratively decide on the appropriateness of surgery for each patient. Intervention patients had half as many major 30-day complications as historical controls. Our proposed case conference nudge is generally similar, but differs in that surgeons’ participation would be optional, proceedings would leverage existing appropriateness criteria, and a searchable database would make conference deliberations available for reference [77, 78].

Our work has several limitations. As would be expected of a Stage I effort to develop a behavioral intervention, we only included a small number of surgeons from two sites in the same geographic area. However, the institutional structures and cultures were distinct, and we sought input from a wide range of stakeholders and experts outside of the study sites. Developing nudge prototypes involved subjective judgments and we did not present all nudge frameworks during the focus groups, emphasizing nudge frameworks where implementation strategies were clearer and excluding those poorly suited to complex decision making. We designed the preop check after the focus groups, reducing feedback obtained on this nudge so far. Nonetheless, the surgeons’ ratings about this nudge were favorable overall and comparable to those of the other nudges.

This work is the first step in developing and testing surgical appropriateness nudges for degenerative lumbar scoliosis and spondylolisthesis. Consistent with the NIH Stage Model of Behavioral Intervention Development, subsequent steps include preparing the nudges for implementation, actually implementing them at these study sites, making further refinements, and then conducting early tests of effectiveness at these study sites. Later stages would involve testing effectiveness in diverse settings to maximize external validity as well as dissemination and implementation research [38]. While the current nudge design process largely relied on qualitative methods, work is needed to demonstrate that these nudged can be implemented in practice, followed by a future randomized controlled trial to test whether the surgical appropriateness nudges can shape surgical decision making. If effective at improving surgeon decision making, nudges may secondarily lower major complication rates and improve patient-reported outcomes.

## Supporting information

S1 File Focus Group 1 results. (DOCX)

S2 File Map of surgical workflow. (DOCX)

S3 File Focus Group 2 results. (DOCX)

S4 File Nudge frameworks considered, inclusion/exclusion, and rationale. (DOCX)

S5 File Refined nudge prototypes: Details on nudge design and examples. (DOCX)

S6 File Pilot testing of refined nudge prototypes: Survey items and results. (DOCX)

S7 File Survey data file. (XLSX)

## References

[1] Debono B, Lonjon G, Galovich LA, Kerever S, Guiot B, Eicker SO, et al. Indication Variability in Degenerative Lumbar Spine Surgery: A Four-nation Survey. Spine (Phila Pa 1976). 2018;43(3):185–92. doi: 10.1097/BRS.0000000000002272 28604486

[2] Irwin ZN, Hilibrand A, Gustavel M, McLain R, Shaffer W, Myers M, et al. Variation in surgical decision making for degenerative spinal disorders. Part I: lumbar spine. Spine. 2005;30(19):2208–13. doi: 10.1097/01.brs.0000181057.60012.08 16205348

[3] Lubelski D, Alentado VJ, Williams SK, O’Rourke C, Obuchowski NA, Wang JC, et al. Variability in Surgical Treatment of Spondylolisthesis Among Spine Surgeons. World neurosurgery. 2018;111:e564–e72. doi: 10.1016/j.wneu.2017.12.108 29288862

[4] Lubelski D, Williams SK, O’Rourke C, Obuchowski NA, Wang JC, Steinmetz MP, et al. Differences in the Surgical Treatment of Lower Back Pain Among Spine Surgeons in the United States. 2016;41(11):978–86. 10.1097/BRS.0000000000001396 26679881

[5] Schroeder GD, Kepler CK, Kurd MF, Vaccaro AR, Hsu WK, Patel AA, et al. Rationale for the Surgical Treatment of Lumbar Degenerative Spondylolisthesis. Spine (Phila Pa 1976). 2015;40(21):E1161–6. doi: 10.1097/BRS.0000000000001116 26274525

[6] Weinstein JN, Lurie JD, Olson PR, Bronner KK, Fisher ES. United States’ trends and regional variations in lumbar spine surgery: 1992–2003. Spine (Phila Pa 1976). 2006;31(23):2707–14. doi: 10.1097/01.brs.0000248132.15231.fe 17077740 PMC2913862

[7] Martin BI, Tosteson AN, Lurie JD, Mirza SK. Variation in the Care of Surgical Conditions: Spinal Stenosis. A Dartmouth Atlas of Health Care Series. Dartmouth Institute; 2014. 36454937

[8] Cook C, Santos GC, Lima R, Pietrobon R, Jacobs DO, Richardson W. Geographic variation in lumbar fusion for degenerative disorders: 1990 to 2000. Spine J. 2007;7(5):552–7. doi: 10.1016/j.spinee.2006.09.010 17905317

[9] Azad TD, Vail D, O’Connell C, Han SS, Veeravagu A, Ratliff JK. Geographic variation in the surgical management of lumbar spondylolisthesis: characterizing practice patterns and outcomes. Spine J. 2018;18(12):2232–8. doi: 10.1016/j.spinee.2018.05.008 29746964

[10] Deyo RA, Mirza SK, Martin BI, Kreuter W, Goodman DC, Jarvik JG. Trends, major medical complications, and charges associated with surgery for lumbar spinal stenosis in older adults. Jama. 2010;303(13):1259–65. doi: 10.1001/jama.2010.338 20371784 PMC2885954

[11] Shekelle P. The appropriateness method. Med Decis Making. 2004;24(2):228–31. doi: 10.1177/0272989X04264212 15090107

[12] Fitch K, Bernstein SJ, Aguilar MD, Burnand B, LaCalle JR, Lazaro P, et al. The RAND/UCLA Appropriateness Method User’s Manual. Santa Monica, CA: RAND Corporation; 2001. Report No.: MR-1269-DG-XII/RE.

[13] Higashi T, Shekelle PG, Adams JL, Kamberg CJ, Roth CP, Solomon DH, et al. Quality of care is associated with survival in vulnerable older patients. Ann Intern Med. 2005;143(4):274–81. doi: 10.7326/0003-4819-143-4-200508160-00008 16103471

[14] Katz JN, Winter AR, Hawker G. Measures of the Appropriateness of Elective Orthopaedic Joint and Spine Procedures. J Bone Joint Surg Am. 2017;99(4). doi: 10.2106/JBJS.16.00473 28196043

[15] Quintana JM, Escobar A, Arostegui I, Bilbao A, Azkarate J, Goenaga JI, et al. Health-related quality of life and appropriateness of knee or hip joint replacement. Arch Intern Med. 2006;166(2):220–26. doi: 10.1001/archinte.166.2.220 16432092

[16] Shekelle PG, Chassin MR, Park RE. Assessing the Predictive Validity of the RAND/UCLA Appropriateness Method Criteria for Performing Carotid Endarterectomy. Int J Technol Assess Health Care. 1998;14(4):707–27. doi: 10.1017/s0266462300012022 9885461

[17] Shekelle PG, Park RE, Kahan JP, Leape LL, Kamberg CJ, Bernstein SJ. Sensitivity and specificity of the RAND/UCLA Appropriateness Method to identify the overuse and underuse of coronary revascularization and hysterectomy. J Clin Epidemiol. 2001;54(10):1004–10. doi: 10.1016/s0895-4356(01)00365-1 11576811

[18] American Academy of Orthopaedic Surgeons. Appropriate Use Criteria. 2017.

[19] American Association of Orthopaedic Surgeons. APPROPRIATE USE CRITERIA: SRS2 2018 [Available from: https://aaos.webauthor.com/go/auc/terms.cfm?auc_id=224992&actionxm=Terms

[20] Cabana MD, Rand CS, Powe NR, Wu AW, Wilson MH, Abboud PA, et al. Why don’t physicians follow clinical practice guidelines? A framework for improvement. Jama Intern Med. 1999;282(15):1458–65. doi: 10.1001/jama.282.15.1458 10535437

[21] Last BS, Buttenheim AM, Timon CE, Mitra N, Beidas RS. Systematic review of clinician-directed nudges in healthcare contexts. Bmj Open. 2021;11(7):e048801. doi: 10.1136/bmjopen-2021-048801 34253672 PMC8276299

[22] Thaler RH, Sunstein CR. Nudge: Improving Decisions About Health, Wealth, and Happiness. New York, NY: Penguin Books; 2009 February 24.

[23] Nagtegaal R, Tummers L, Noordegraaf M, Bekkers V. Nudging healthcare professionals towards evidence-based medicine: a systematic scoping review. Journal of Behavioral Public Administration. 2019;2(2).

[24] Nwafor O, Singh R, Collier C, DeLeon D, Osborne J, DeYoung J. Effectiveness of nudges as a tool to promote adherence to guidelines in healthcare and their organizational implications: A systematic review. Soc Sci Med. 2021;286:114321. doi: 10.1016/j.socscimed.2021.114321 34438185

[25] Sant’Anna A, Vilhelmsson A, Wolf A. Nudging healthcare professionals in clinical settings: a scoping review of the literature. BMC Health Serv Res. 2021;21(1):543. doi: 10.1186/s12913-021-06496-z 34078358 PMC8170624

[26] Yoong SL, Hall A, Stacey F, Grady A, Sutherland R, Wyse R, et al. Nudge strategies to improve healthcare providers’ implementation of evidence-based guidelines, policies and practices: a systematic review of trials included within Cochrane systematic reviews. Implement Sci. 2020;15(1):50. doi: 10.1186/s13012-020-01011-0 32611354 PMC7329401

[27] Hare AJ, Patel MS, Volpp K, Adusumalli S. The Role of Behavioral Economics in Improving Cardiovascular Health Behaviors and Outcomes. Curr Cardiol Rep. 2021;23(11):153. doi: 10.1007/s11886-021-01584-2 34599461 PMC8485972

[28] Wang SY, Groene O. The effectiveness of behavioral economics-informed interventions on physician behavioral change: A systematic literature review. PLoS One. 2020;15(6):e0234149. doi: 10.1371/journal.pone.0234149 32497082 PMC7272062

[29] Talat U, Schmidtke KA, Khanal S, Chan A, Turner A, Horne R, et al. A Systematic Review of Nudge Interventions to Optimize Medication Prescribing. Front Pharmacol. 2022;13:798916. doi: 10.3389/fphar.2022.798916 35145411 PMC8822212

[30] Cho I, Bates DW. Behavioral Economics Interventions in Clinical Decision Support Systems. Yearb Med Inform. 2018;27(1):114–21. doi: 10.1055/s-0038-1641221 30157514 PMC6115210

[31] Khullar D. How Behavioral Economics Can Produce Better Health Care. New York Times. 2017 April 13.

[32] Loewenstein G, Brennan T, Volpp KG. Asymmetric paternalism to improve health behaviors. Jama Intern Med. 2007;298(20):2415–7. doi: 10.1001/jama.298.20.2415 18042920

[33] Scheiber N. How Uber Uses Psychological Tricks to Push Its Drivers’ Buttons. New York Times. 2017 April 2.

[34] Thaler R. The Power of Nudges, for Good and Bad. The New York Times. 2015 October 31.

[35] Samson A. The Behavioral Economics Guide. 2016.

[36] Mannion AF, Pittet V, Steiger F, Vader JP, Becker HJ, Porchet F, et al. Development of appropriateness criteria for the surgical treatment of symptomatic lumbar degenerative spondylolisthesis (LDS). Eur Spine J. 2014;23(9):1903–17. doi: 10.1007/s00586-014-3284-0 24760463

[37] Chen PGC, Daubs MD, Berven S, Raaen LB, Anderson AT, Asch SM, et al. Surgery for Degenerative Lumbar Scoliosis: The Development of Appropriateness Criteria. Spine. 2016;41(10):910–8. doi: 10.1097/BRS.0000000000001392 26679874

[38] National Institute on Aging. NIH Stage Model for Behavioral Intervention Development [Available from: https://www.nia.nih.gov/research/dbsr/nih-stage-model-behavioral-intervention-development.

[39] Hawkins J, Madden K, Fletcher A, Midgley L, Grant A, Cox G, et al. Development of a framework for the co-production and prototyping of public health interventions. BMC Public Health. 2017;17(1):689. doi: 10.1186/s12889-017-4695-8 28870192 PMC5583990

[40] Czajkowski SM, Powell LH, Adler N, Naar-King S, Reynolds KD, Hunter CM, et al. From ideas to efficacy: The ORBIT model for developing behavioral treatments for chronic diseases. Health Psychol. 2015;34(10):971–82. doi: 10.1037/hea0000161 25642841 PMC4522392

[41] Malterud K. The art and science of clinical knowledge: evidence beyond measures and numbers. Lancet. 2001;358(9279):397–400. doi: 10.1016/S0140-6736(01)05548-9 11502338

[42] Patton MQ. Qualitative Research & Evaluation Methods 3rd edition ed: Sage Publications, Inc.; 2002.

[43] Maudsley G. Mixing it but not mixed-up: mixed methods research in medical education (a critical narrative review). Med Teach. 2011;33(2):e92–104. doi: 10.3109/0142159X.2011.542523 21275539

[44] Whittemore R, Chase SK, Mandle CL. Validity in qualitative research. Qual Health Res. 2001;11(4):522–37. doi: 10.1177/104973201129119299 11521609

[45] O’Brien BC, Harris IB, Beckman TJ, Reed DA, Cook DA. Standards for Reporting Qualitative Research: A Synthesis of Recommendations. Academic Medicine. 2014;89(9):1245–51. doi: 10.1097/ACM.0000000000000388 24979285

[46] Bourgeault I, Dingwall R, De Vries R. The SAGE Handbook of Qualitative Methods in Health Research. London 2010. Available from: https://methods.sagepub.com/book/sage-hdbk-qualitative-methods-in-health-research.

[47] Sofaer S. Qualitative methods: what are they and why use them? Health services research. 1999;34(5 Pt 2):1101–18. 10591275 PMC1089055

[48] Bradley EH, Curry LA, Devers KJ. Qualitative data analysis for health services research: developing taxonomy, themes, and theory. Health services research. 2007;42(4):1758–72. doi: 10.1111/j.1475-6773.2006.00684.x 17286625 PMC1955280

[49] Mannion AF, Fekete TF, Porchet F, Haschtmann D, Jeszenszky D, Kleinstuck FS. The influence of comorbidity on the risks and benefits of spine surgery for degenerative lumbar disorders. Eur Spine J. 2014;23 Suppl 1:S66–71. doi: 10.1007/s00586-014-3189-y 24458936 PMC3946098

[50] Daubs MD BH, Raaen LB, Chen PG, Anderson AT, Asch SM, Nuckols TK. How does sagittal imbalance affect the appropriateness of surgical indications and selection of procedure in the treatment of degenerative scoliosis? Findings from the RAND/UCLA Appropriate Use Criteria study Spine J. 2018;18(5):900–11. 29412187 10.1016/j.spinee.2018.01.027

[51] Hendel Robert C, Lindsay Bruce D, Allen Joseph M, Brindis Ralph G, Patel Manesh R, White L, et al. ACC Appropriate Use Criteria Methodology: 2018 Update. Journal of the American College of Cardiology. 2018;71(8):935–48. 29471942 10.1016/j.jacc.2018.01.007

[52] Lawson EH, Gibbons MM, Ko CY, Shekelle PG. The appropriateness method has acceptable reliability and validity for assessing overuse and underuse of surgical procedures. J Clin Epidemiol. 2012;65(11):1133–43. doi: 10.1016/j.jclinepi.2012.07.002 23017632

[53] Vader JP, Porchet F, Larequi-Lauber T, Dubois RW, Burnand B. Appropriateness of surgery for sciatica – Reliability of guidelines from expert panels. Spine. 2000;25(14):1831–6. doi: 10.1097/00007632-200007150-00015 10888953

[54] Lawson EH, Gibbons MM, Ingraham AM, Shekelle PG, Ko CY. Appropriateness criteria to assess variations in surgical procedure use in the United States. Arch Surg. 2011;146(12):1433–40. doi: 10.1001/archsurg.2011.581 22184308

[55] Shekelle PG, Kahan JP, Bernstein SJ, Leape LL, Kamberg CJ, Park RE. The reproducibility of a method to identify the overuse and underuse of medical procedures. N Engl J Med. 1998;338(26):1888–95. doi: 10.1056/NEJM199806253382607 9637810

[56] Daubs MD, Brara HS, Raaen L, Chen PG, Anderson AT, Asch SM, et al. How Does Sagittal Imbalance Affect the Appropriateness of Surgical Indications and Selection of Procedure in the Treatment of Degenerative Scoliosis?: Findings from the RAND/UCLA Appropriate Use Criteria Study2018. 10.1016/j.spinee.2018.01.027 29412187

[57] American Academy of Orthopaedic Surgeons. Appropriate Use Criteria: Surgery For Degenerative Lumbar Scoliosis. 2016.

[58] Hastie R, Dawes RM. Rational choice in an uncertain world: The psychology of judgment and decision making: Sage Publications; 2009.

[59] Camerer CF, Loewenstein G, Rabin M. Advances in behavioral economics: Princeton university press; 2004.

[60] Kahneman D, Slovic SP, Slovic P, Tversky A. Judgment under uncertainty: Heuristics and biases: Cambridge university press; 1982. 10.1126/science.185.4157.1124 17835457

[61] Tversky A, Kahneman D. Judgment under Uncertainty: Heuristics and Biases: Biases in judgments reveal some heuristics of thinking under uncertainty. science. 1974;185(4157):1124–31. 17835457 10.1126/science.185.4157.1124

[62] Thaler RH. The Power of Nudges, for Good and Bad. New York Times. 2015 11/1/2015.

[63] Krijnen JM, Tannenbaum D, Fox CR. Choice architecture 2.0: Behavioral policy as an implicit social interaction. Behavioral Science & Policy. 2017;3(2):i–18.

[64] Zürich Appropriateness of Spine Surgery (ZASS) Group: Criteria for Lumbar Degen Spondylolisthesis. Appropriate Use Criteria App: American Academy of Orthopaedic Surgeons; 2018 [Available from: http://schulthess.webauthor.com/go/auc/.

[65] Shetty KD, Chen PG, Brara HS, Anand N, Skaggs DL, Calsavara VF, et al. Variations in Surgical Practice and Short-term Outcomes for Degenerative Lumbar Scoliosis and Spondylolisthesis: Do Surgeon Training and Experience Matter? Int J Qual Health Care. 2023. 10.1093/intqhc/mzad109 PMC10849168 38156345

[66] Weiner BJ, Lewis CC, Stanick C, Powell BJ, Dorsey CN, Clary AS, et al. Psychometric assessment of three newly developed implementation outcome measures. Implement Sci. 2017;12(1):108. doi: 10.1186/s13012-017-0635-3 28851459 PMC5576104

[67] Mannion AF, Mariaux F, Pittet V, Steiger F, Aepli M, Fekete TF, et al. Association between the appropriateness of surgery, according to appropriate use criteria, and patient-rated outcomes after surgery for lumbar degenerative spondylolisthesis. Eur Spine J. 2021;30(4):907–17. doi: 10.1007/s00586-021-06725-3 33575818

[68] Jacobs E, van Kuijk SMJ, Merk JMR, Vandewall-Peeters M, Jütten-Brouwer LMC, van Rhijn LW, et al. Implementation of patient-reported outcome measures in appropriateness criteria of surgery for degenerative lumbar scoliosis. The Spine Journal. 2019;19(4):655–61. doi: 10.1016/j.spinee.2018.09.012 30261263

[69] Emanuel EJ, Ubel PA, Kessler JB, Meyer G, Muller RW, Navathe AS, et al. Using Behavioral Economics to Design Physician Incentives That Deliver High-Value Care. Ann Intern Med. 2016;164(2):114–9. doi: 10.7326/M15-1330 26595370

[70] Reed KL, Harvey EM, Everly CJ. The Intersection of Behavioral Economics and the General Medicine Literature. Am J Med. 2021;134(11):1350–6.e2. doi: 10.1016/j.amjmed.2021.06.041 34343511

[71] O’Keeffe M, Traeger AC, Hoffmann T, Ferreira GE, Soon J, Maher C. Can nudge-interventions address health service overuse and underuse? Protocol for a systematic review. Bmj Open. 2019;9(6):e029540. doi: 10.1136/bmjopen-2019-029540 31239308 PMC6597741

[72] Brehaut JC, Colquhoun HL, Eva KW, Carroll K, Sales A, Michie S, et al. Practice Feedback Interventions: 15 Suggestions for Optimizing Effectiveness. Ann Intern Med. 2016;164(6):435–41. doi: 10.7326/M15-2248 26903136

[73] National Academies of Sciences E, and Medicine,. Behavioral Economics and the Promotion of Health Among Aging Populations: Proceedings of a Workshop—in Brief. Washington, DC: The National Academies Press; 2018. 30024689

[74] Dolan P, Hallsworth M, Halpern D, King D, Vlaev I. MINDSPACE: influencing behaviour for public policy. 2010.

[75] Cialdini RB, Reno RR, Kallgren CA. A focus theory of normative conduct: Recycling the concept of norms to reduce littering in public places. Journal of personality and social psychology. 1990;58(6):1015.

[76] Kallgren CA, Reno RR, Cialdini RB. A focus theory of normative conduct: When norms do and do not affect behavior. Personality and social psychology bulletin. 2000;26(8):1002–12.

[77] Sethi R, Buchlak QD, Yanamadala V, Anderson ML, Baldwin EA, Mecklenburg RS, et al. A systematic multidisciplinary initiative for reducing the risk of complications in adult scoliosis surgery. Journal of neurosurgery Spine. 2017;26(6):744–50. doi: 10.3171/2016.11.SPINE16537 28362214

[78] Buchlak QD, Yanamadala V, Leveque JC, Sethi R. Complication avoidance with pre-operative screening: insights from the Seattle spine team. Curr Rev Musculoskelet Med. 2016;9(3):316–26. doi: 10.1007/s12178-016-9351-x 27260267 PMC4958383

