# Supplementary material for: Surgical appropriateness nudges: Developing behavioral science nudges to integrate appropriateness criteria into the decision making of spine surgeons
Source: PLoS One. 2024 Apr 19;19(4):e0300475. doi: 10.1371/journal.pone.0300475 (PMC11029649; doi:10.1371/journal.pone.0300475)
Supplement: S1 File — (DOCX) [file pone.0300475.s001.docx]

**S1 File: Focus Group 1 Results**

| **Topic** | **Surgeon responses** | **Illustrative quotes** |
| --- | --- | --- |
| System-level variation | Some institutions impose requirements that patients under consideration for major spine surgery must undergo smoking cessation, osteoporosis treatment or psychosocial evaluation to address existing issues prior to undergoing spine surgery.  Some institutions may not have required ancillary services, thereby eliminating the possibility of some surgical approaches.  Some institutions have committees to decide on purchasing devices and other equipment, while other institutions defer to individual surgeon preferences.  Financial pressures related to reimbursement and revenue generation, could influence variation in surgery. | “Some [surgical] choices depend on the availability of vascular surgery to make an anterior approach to surgery safe.”  “There is some value in institutional pressures where costs and availability [place limits on the potential overuse of] procedures.”  “Reimbursement is the elephant in the room. Not only consciously but subconsciously.”  “The industry influence is significant. Every meeting there are vendors showing off implants.” |
| Surgeon level variation | Surgeons identified training, career phase and individual experiences as important sources of variation.  Individual surgeons’ habits evolve over time and based on experiences and career phase.  Surgeons did not generally feel that there were systematic differences between orthopedic-trained and neurosurgery-trained spine surgeons though they did identify changes in decision making over a surgeon’s career. | “Unlike in other fields, there is no Level I evidence for anything we do in term of guidelines. A lot is influenced by who, where and when you trained.”  “There is an age dynamic. When you are young, you fuse. Middle career, you re-fuse your fusions, and when you are old you refuse to fuse.”  “Thinking about how my career has evolved, I tended to treat x-rays and imaging more earlier, but then I moved to symptoms dictating my choices.”  “If you have a bad outcome, that can influence you as well. You get clouded and make different decisions in the future.”  “Depending on the surgeon, some are early adopters and some are wait and see.” |
| Patient level variation | General consensus that factors such as age, comorbidities and alignment parameters play an important role.  Patient out-of-pocket costs are an important factor.  Patient preferences, expectations and goals are important. | “Some approaches may be more expensive to patients than others, vascular surgeons for anterior approach may not be covered by insurance, for example, which makes patients choose other procedures.”  “Consider duration of relief – if patients feel better for 2-3 years, is that enough? Is that a success or a failure? Depends on the patient.” |
| Spine surgeon workflow | At both study sites, patients were generally referred to spine surgeons after conservative medical management failed, and after most imaging studies including MRI and X-ray were conducted.  Initial surgical consultation requires a good history and physical for effective decision-making, including eliciting a clear understanding of patient goals. Most decision-making by the surgeon occurred at this stage, followed by patients weighing options given to them by one or more surgeons.  Potential surgical options start out broad, and are whittled down as more information (e.g. clinical and radiographic data) as well as patient factors including patient goals, expectations, and preferences.  Patients are referred for surgery after mutual agreement by both the patient and surgeon, and there was no formal review in place either before or after the operation although cases could be flagged before or after the surgery.  Major decisions were made prior to taking the patient to the operating room, but minor adjustments are often made intra-operatively.  Some divergent practices between sites: Cedars Sinai, has little gatekeeping and surgeons accept referrals from all types of providers. The review process, although not sought in most cases, includes educational conferences in which cases are discussed with residents, fellows, and attending surgeons.  Kaiser Permanente, with the exception of urgent cases (e.g., cauda equina), referrals need to come from designated specialties: neurosurgery, orthopedics, physical medicine and rehabilitation, pain management, neurology, and spine clinic. Patients often choose to have second opinions but visits for 2^nd^ opinions are generally shorter (20 minutes in comparison to one hour for new consults). | “The first step [is] where you look at films and see the referral. I am synthesizing what are the options available. Once I am in the room, it goes back to the shared decision that we discuss. We give options and risk and benefits. We make the decisions after that. There is variation throughout, even in data gathering.”  “In surgery, you may do one less interbody. I would not change a plan majorly mid-surgery… unless there is a complication I typically do not deviate from the plan.” |

**Synthesis of Existing Information: “Maps” of Preoperative Workflows at Two Regional Referral Centers for Spine Surgery**

| **Institution** | **Referral 🡪** | **Surgeon Assess Risks/Benefits 🡪** | **Surgeon Discusses with Patient 🡪** | **Quality Assurance Procedures 🡪** | **Intra-operative Decision-making** |
| --- | --- | --- | --- | --- | --- |
| **Common to both institutions** | • Studies (MRI, X-ray) usually completed in advance • Prior conservative management failed | • Good history & physical critical  • Elicit patient goals  • Order additional studies as needed  • Nearly all decision-making about whether to offer surgery (and which procedure) occurs at this stage  • Data for applying AUC not available in electronic format | • Patients weigh options • Patients seek second opinion if desired | • Patient scheduled for surgery after agreement by patient and surgeon • No formal review by department or hospital • Informal case review sought from colleagues | • Typically, no major changes to procedure choice or approach immediately before or during procedure • No formal feedback mechanism outside of mortality and morbidity review |
| **Site 1 only** | • Referral accepted from all sources, including primary care | • Process for seeing patients not standardized between surgeons |  | • Educational conferences discuss cases with residents and fellows (ad hoc) |  |
| **Site 2 only** | • Referrals accepted from specialists (neurosurgery, orthopedics, physical medicine and rehabilitation, pain management, neurology, or specialized spine clinic) | • Surgeons given up to 1 hour for new consults  • Surgeons are given 20 minutes for visits for second opinions  • Order additional studies as needed but most studies are readily available through electronic health record  • Patients generally receive three options, although some patients with more co-morbidities may only receive less-invasive options | • Patient given 2 weeks to weigh options • Second opinion often sought when surgery not offered or when recommended procedure more invasive than desired by patient | • New technologies are assessed by central Medical Technology Assessment Team (MTAT) team • Surgeons may seek assistance on complex cases |  |

**Focus Group 2: Results**

| **Nudge Type** | **Surgeon responses** | **Illustrative quotes** |
| --- | --- | --- |
| Structured note template | If it helps facilitate efficiency and completeness of documentation, it would garner high levels of buy-in  In systems with open notes, patients may review their providers’ clinical notes. The structured note template could help patients have more confidence in the ultimate surgical decision  Ease of use is vital.  Documentation practices can be highly variable which might make structured note template challenging. Some surgeons rely on spine fellows, rotating residents, and/or physicians assistants to document clinic notes; other surgeons use dictation or voice recognition software to generate clinic notes. | “Any approach for minimizing work would get buy-in quickly. For instance, an EPIC template that could pull in data quickly and captures data surgeons need to have anyway.”  “[While it’s] important to adopt standardized dot phrases to streamline workflow… [it] has to be easy to use and flexible because surgical decision-making is nuanced.” |
| Online calculator | Surgeons already bookmark online calculators for other conditions and use them frequently when the clinical situation is appropriate.  Existing calculators could be augmented to be even more useful to surgeons. | “A phrase that would pop up after using the calculator online would be useful because surgeons could paste the phrase into their note. Something like ‘I used the AAOS DLS Appropriateness Calculator which rated patients with this clinical scenario as appropriate for surgical intervention.’ with a reference, could help justify surgeon’s decision making and be pasted into the clinical note.” |
| Multispecialty conferences | Surgeons noted they really need additional support in scenarios for which the appropriateness method does not make a recommendation on appropriateness or inappropriateness. Multispecialty conferences are already fairly common in surgical training.  Multispecialty conference should be optional and not required.  Important to set ground rules so that all participants have equal opportunities to contribute. | “The presence of a few strong personalities might stifle open discussion.”  Without careful planning, such a conference could also “homogenize to the institution’s culture” (e.g. result in regression to the mean).  Group size for such conference is an important consideration since groups that are too large often mean that “decisions don’t get made”. |
| Accountable justification | Surgeons receptive to accountable justification, but did not have strong feelings or discuss this type of nudge in great detail. | “It could be useful, it’s nice to have a flag for providing the rationale for a procedure.”  Accountable justification could “help to track behavior”. |
| Individualized score card | **Individualized score card** received mixed reviews from surgeons.  If the score card is used for public shaming, it could be problematic.  Concerns that the score cards could be used punitively (e.g., to decrease operating room time).  Scoring approach must be transparent and logical. Patient case identification should be shared because ICD-10 codes may not always be accurately applied.  Several surgeons were very interested to see how their practices and outcomes compared with peers, particularly their closest peers within their own practice groups. | “Maybe high performer public reporting would be good, but individual results should not be publicly shared.” |

**Nudge Frameworks Considered, Ultimate Inclusion/Exclusion, and Rationale**

| Nudge Framework | Example from Setting in Healthcare | Inclusion/Exclusion in Refined Nudge Prototypes | Rationale |
| --- | --- | --- | --- |
| **Nudge Frameworks Included in Preliminary Nudge Prototypes** | | | |
| **Peer comparisons:** Providing individuals with data on their performance relative to similar peers; references descriptive norms | Comparing physician antibiotic prescribing rates with those of “top performers.” (Meeker et al., 2016; Linder et al., 2017) | *Yes, included in Individualized Score Cards* | Surgeons value but do not consistently receive information about their performance. They value most highly data on peers within their own practices. |
| **Descriptive norm:** Reflects how people actually behave in practice. | Same as above | *Yes, included in Individualized Score Cards* | Same as above |
| **Feedback:** Reporting the frequency or occurrence of the target behavior back to the user | Same as above | *Yes, included in Individualized Score Cards* | Some surgeons are accustomed to score cards and would appreciate additional feedback to support improvement. |
| **Framing:** People pay more attention to negative vs. positive information | Physicians prefer a riskier treatment when outcomes are presented as lives lost rather than lives saved (Almashat et al. 2008) | *Relevant to Individualized Score Cards, but ultimately excluded* | Feedback to clinicians should be presented in non-judgmental, constructive way to avoid triggering defensive reactions. |
| **Decision Aid:** Tools that help with considering several different options | Tools to help with deciding about stool testing in colorectal cancer screening (Schwartz et al. 2017) | *Yes, included in Online Calculator* | Surgeons felt appropriateness calculators could complement many other nudge frameworks as well as be used on their own. |
| **Mapping:** Simplifying information about the relationship between choice alternatives and the resulting  outcomes | Emphasizing implications of hand hygiene for patients hygiene (Grant and Hofmann, 2011) | *Yes, included in Online Calculator* | Employing simple symbols and colors draws attention to the anticipated risks vs. benefits of surgical options. |
| **Injunctive Social Norm:** Presenting a desired decision as common and “normal” behavior | Health care team leaders led activities in their units to encourage hand hygiene (Huis et al., 2012) | *Yes, included in Multispecialty Case Conference* | Surgeons value the opinions of thought leaders in their fields and want to “do well”. |
| **Reminders/Alerts:** Calling people’s attention to a desired behavior with timely message | Hospital staff received reminders/alerts for annual influenza vaccination (Schmidtke et al. 2019) | *Yes, included in Preoperative Check* | Surgeons appreciated the additional support to being able to provide explanation for why certain aspects of surgical decision-making may not fall within strictly defined criteria. |
| **Salience of Information:** Presenting information relevant to a decision in a way that enhances its prominence or visibility | Present information on costs to physicians at the time of test ordering to avoid unnecessary testing (Sedrak et al., 2017) | *Yes, included in Preoperative Check* | Providing information tailored to individual patients before surgery may have the greatest potential to shape surgeon behavior. |
| **Accountable justification:** Asking individuals to document rationale for making less desired decisions. | Electronically prompting clinicians to document justifications for potentially inappropriate antibiotics (Meeker et al., 2016) | *Yes, included in Preoperative Check* | Surgeons welcomed an opportunity to explain why certain aspects of surgical decision-making do not fall within strictly defined criteria. |
| **Defaults:** A particular choice is ‘preset’, making it the easiest option | Setting prescription drugs to default to generic equivalents (Malhotra et al., 2016) | *Relates to the Structured Note Template discussed in Focus Group 2, but ultimately excluded* | Documentation practices are too highly variable to rely on a structured note template. Recommended care varies greatly based on patient characteristics, and this makes other applications of defaults risky. |
| **Common Nudge Frameworks Excluded from Preliminary Nudge Prototypes:** These nudges were challenging to apply to complex decision making where there are multiple common surgical procedures that can be used, and recommended care differs greatly across patients. | | | |
| **Priming:** Create physical, verbal or sensational cues that subconsciously shift participants toward a particular choice | Placing hand sanitizer dispenser to improve hand hygiene among anesthesiology staff in the operating room (Munoz-Price et al. 2014) | *No, excluded* | Choosing a surgical procedure for an individual patient is an active not subconscious choice. |
| **Menu partition:** Splitting potential options into groups to facilitate selection of a recommended option | Listing narrow-spectrum antibiotics individually while grouping broad-spectrum antibiotics together (Tannenbaum et al., 2015) | *No, excluded* | Surgeons’ notes are often not completed until after the patient visit, creating mismatch with the need to present surgical options in real-time. |
| **Anchors:** Initial exposure to a number serves as a reference point and influences decisions | E-prescribing platforms automatically set new opioid prescriptions to 10 tablets (Delgado et al., 2018) | *No, excluded* | Numbers are not relevant to the selection of a surgical procedure. |
| **Pre-commitment:** Asking individuals to commit to a decision in advance of actually making the decision | Asking physicians to post exam room signs committing to appropriate antibiotic prescribing (Meeker et al., 2014) | *No, excluded* | Framework assumes the decision is straightforward and easy to identify and adhere to. Surgical decision making is more complex and subject to interpretation so it’s hard for surgeons to meaningfully make a pre-commitment. |

**Refined Nudge Prototypes**

Online Calculators: See text and figure in text for description of the calculators and example of an application to a hypothetical patient.

Individualized Surgeon Score cards: We developed a model score card that presented data on each surgeon’s use of instrumented fusion and rates of major in-hospital complications relative to peers across both study sites, as well as adherence to appropriateness criteria.

First, we created tables and graphics with rates of instrumented fusion and major in-hospital surgical complications for the individual participating surgeon and 88 eligible peers. This involved specifying ICD-10-CM and CPT codes, obtaining administrative data for 2017-2019, identifying 89 eligible surgeons and 2,481 eligible operations, and calculating surgeon-level rates of instrumented fusion as well as major in-hospital complications (hemorrhage, mechanical complications, dural tears, surgical site infections, anesthesia complications, or mortality during the index hospitalization).

Second, the model score cards illustrated how adherence to appropriateness criteria would be reported. For the six participating surgeons and 6 additional surgeons (3 per site) chosen at random, we randomly selected five patients each (60 total). We then trained nurses and physician assistants at study sites to manually review of the selected patients’ medical records, apply additional eligibility criteria specific to the appropriateness criteria, and score adherence to the appropriateness criteria. This produced data on adherence for 2-4 patients per surgeon.

See sample score card for hypothetical spine surgeon below.

In the pilot tests, we created a score card and distributed it to each of the 6 participating surgeons.

Multispecialty Case Conference: See below.

In the pilot tests, we shared this description with the six participating surgeons.

Preoperative Appropriateness Checks (“Preop Checks”): Preop checks would involve using the EHR to detect when surgeons schedule patients for eligible operations, and then emailing the surgeon a set of recommendations based on the appropriateness criteria as well as relevant resources for assessing appropriateness themselves. Each email would outline the patient’s clinical characteristics related to appropriateness, the appropriateness recommendation for the patient, and actions the surgeons could take if the recommendations diverged from the operation they had planned. The patients and data included in the preop checks were the same as those included in the score cards.

See sample preop check for a hypothetical spine surgeon below.

In the pilot tests, we sent two sample preop check emails to each of the six participating surgeons (12 patients total). The patients for whom we presented data were the same as for the individualized score card.

**Refined Nudge Prototype: Individualized Score card for Hypothetical Surgeon**

**Surgical Outcomes and Volume:**

**Period of Time Covered:** January 1, 2017 – December 31, 2019

Number of Operations Reviewed: [N]*

**Part 1: Your Major In-hospital Complication Rate Relative to Other Spine Surgeons:**

| Scoliosis | Spondylolisthesis |
| --- | --- |
| 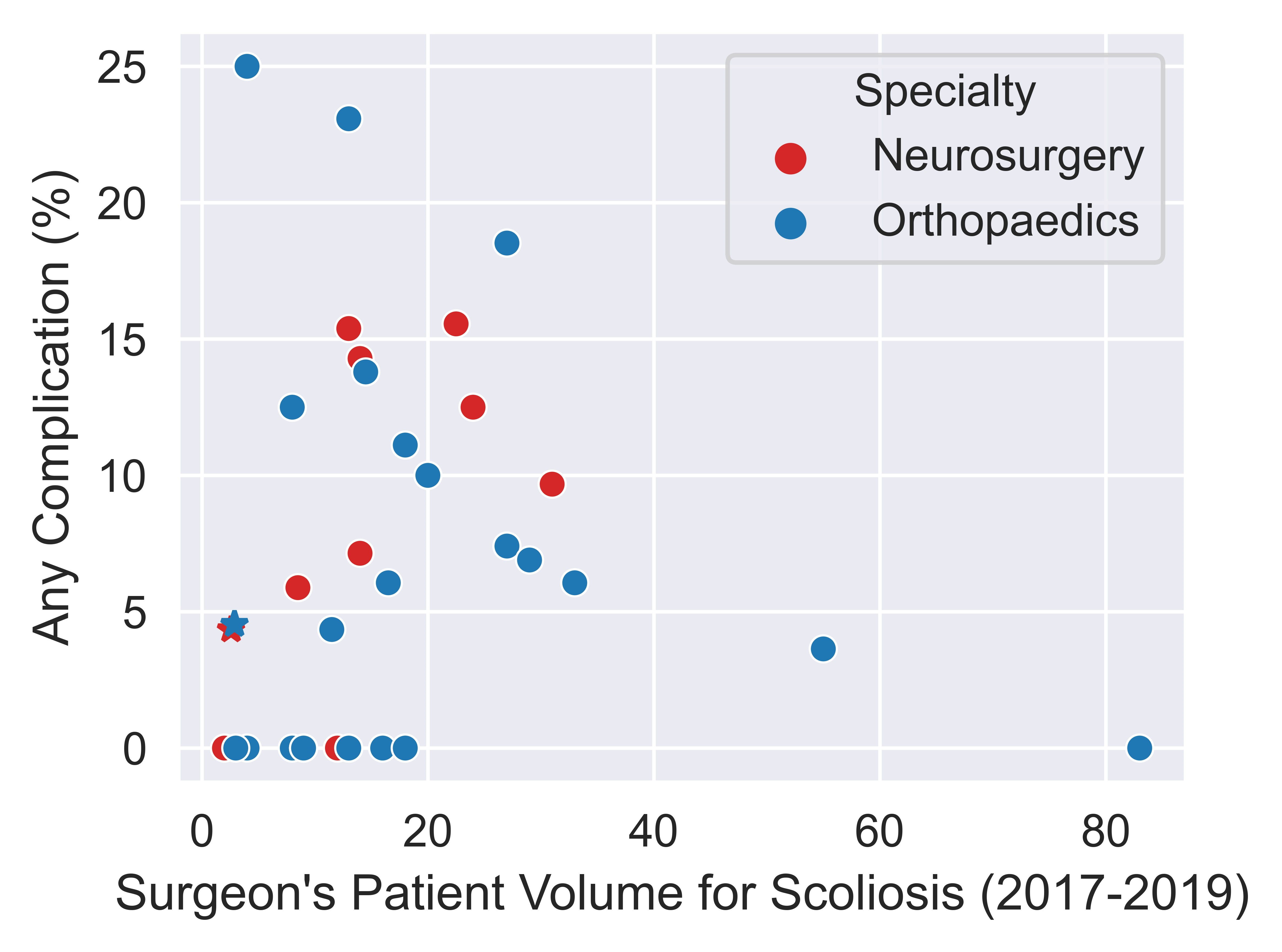 | 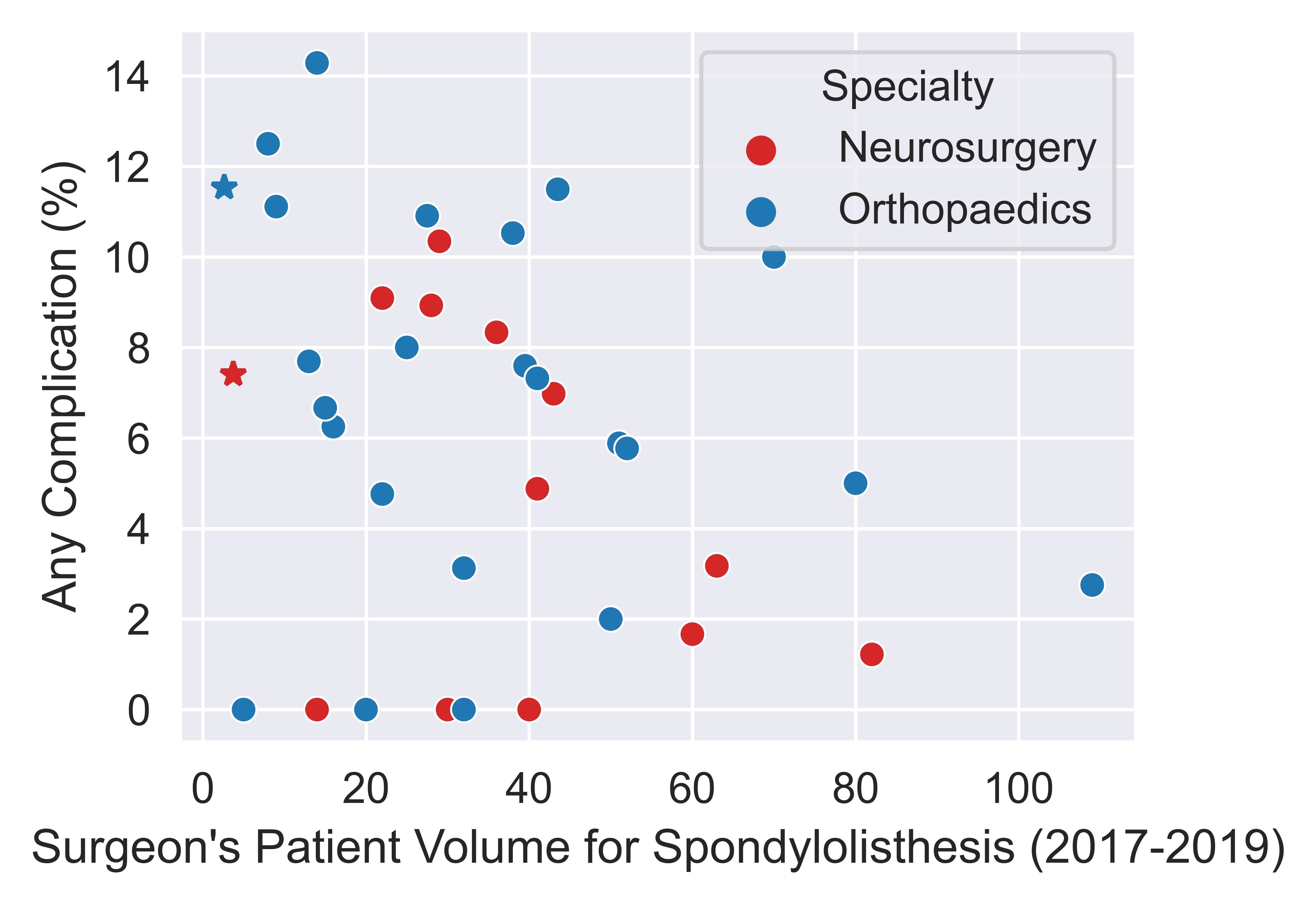 |

Your results are displayed with a red star if 4 or more relevant cases were identified for that diagnosis.

**Summary Table on Complications Relative to Other Spine Surgeons:**

|  | **Rates Among All Surgeons by Percentile** | | | | | **Your Data** | |
| --- | --- | --- | --- | --- | --- | --- | --- |
| **Diagnosis** | **10th** | **25th** | **50th** | **75th** | **90th** | **Your Eligible Cases*** | **Your Rate*** |
| Spondylolisthesis | 0.0 | 0.0 | 0.05 | 0.1 | 0.21 | [N] | X.XX |
| Scoliosis | 0.0 | 0.0 | 0.0 | 0.07 | 0.17 | [N] | X.XX |

Note: Statistics for comparison surgeons represent all spine surgeons at both site 1 (N=42) and site 2 (N=47). *Eligible cases refers to the number of your surgical procedures for each diagnosis that were included in the study. Your rate was computed as the proportion of eligible cases that recorded one or more surgical complications (as defined by ICD-10 codes).

**Specific patients of yours who were identified as having inpatient complications are listed below:**

| **Deidentified Patient MRN** | **Diagnosis** | **Instrumented Fusion** | **Complication(s)** |
| --- | --- | --- | --- |
| [XXXXXXXXXXX] | Spondylolisthesis | Yes | [From list below] |
| [XXXXXXXXXXX] | Scoliosis | Yes | [From list below] |
| [XXXXXXXXXXX] | Scoliosis | No | [From list below] |

* All surgical cases were identified from electronic health record system at your institution based on International Classification of Diseases, 10^th^ edition (ICD-10) and Current Procedural Terminology (CPT) codes. Complications were identified using ICD-10 codes. Major in-hospital complications included: hemorrhage, mechanical complications, dural tears, surgical site infections, anesthesia complications, or mortality during the index hospitalization.

**Part 2: Your Use of Instrumented Fusion Relative to Other Spine Surgeons:**

| Scoliosis | Spondylolisthesis |
| --- | --- |
| 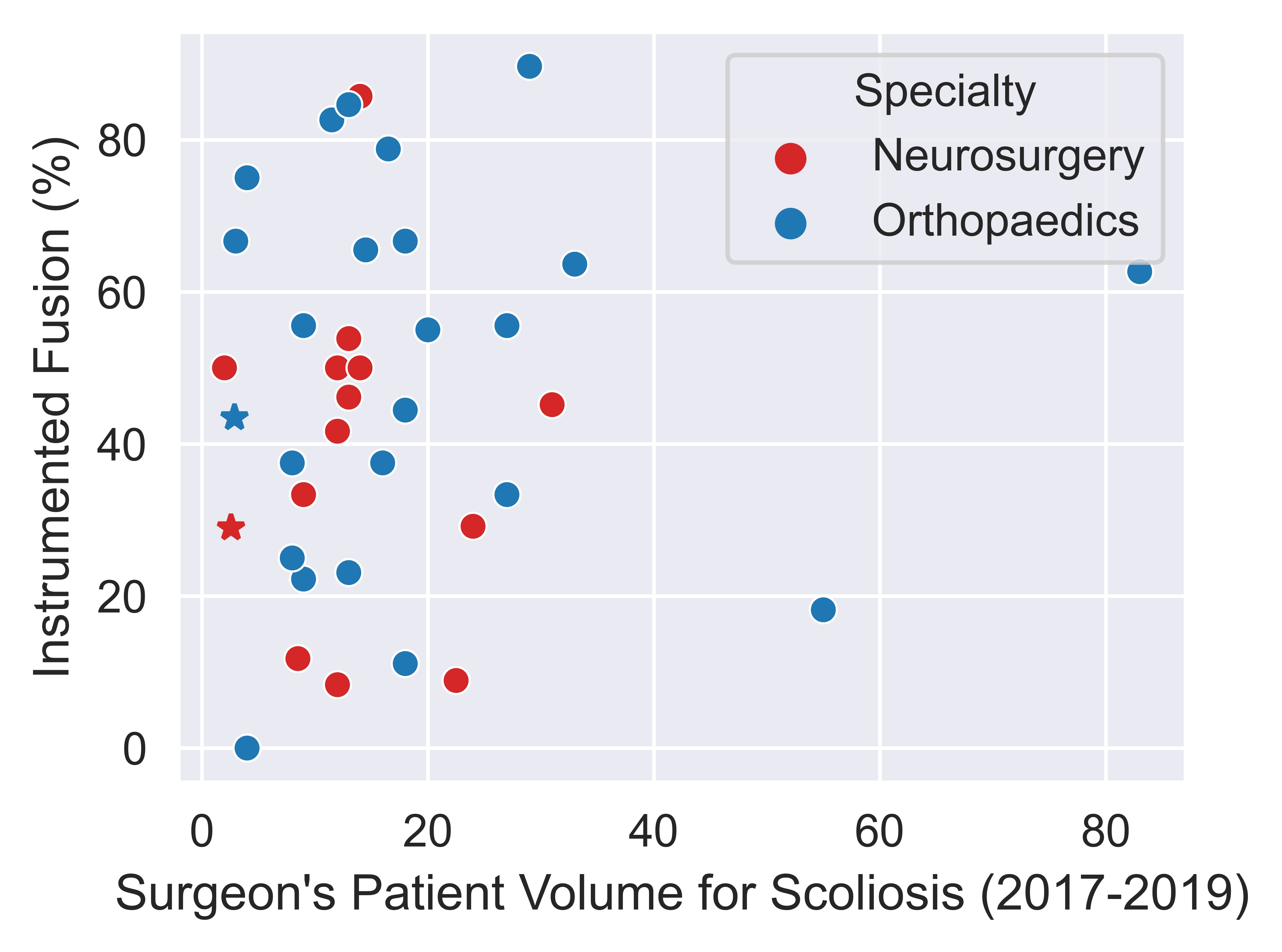 | 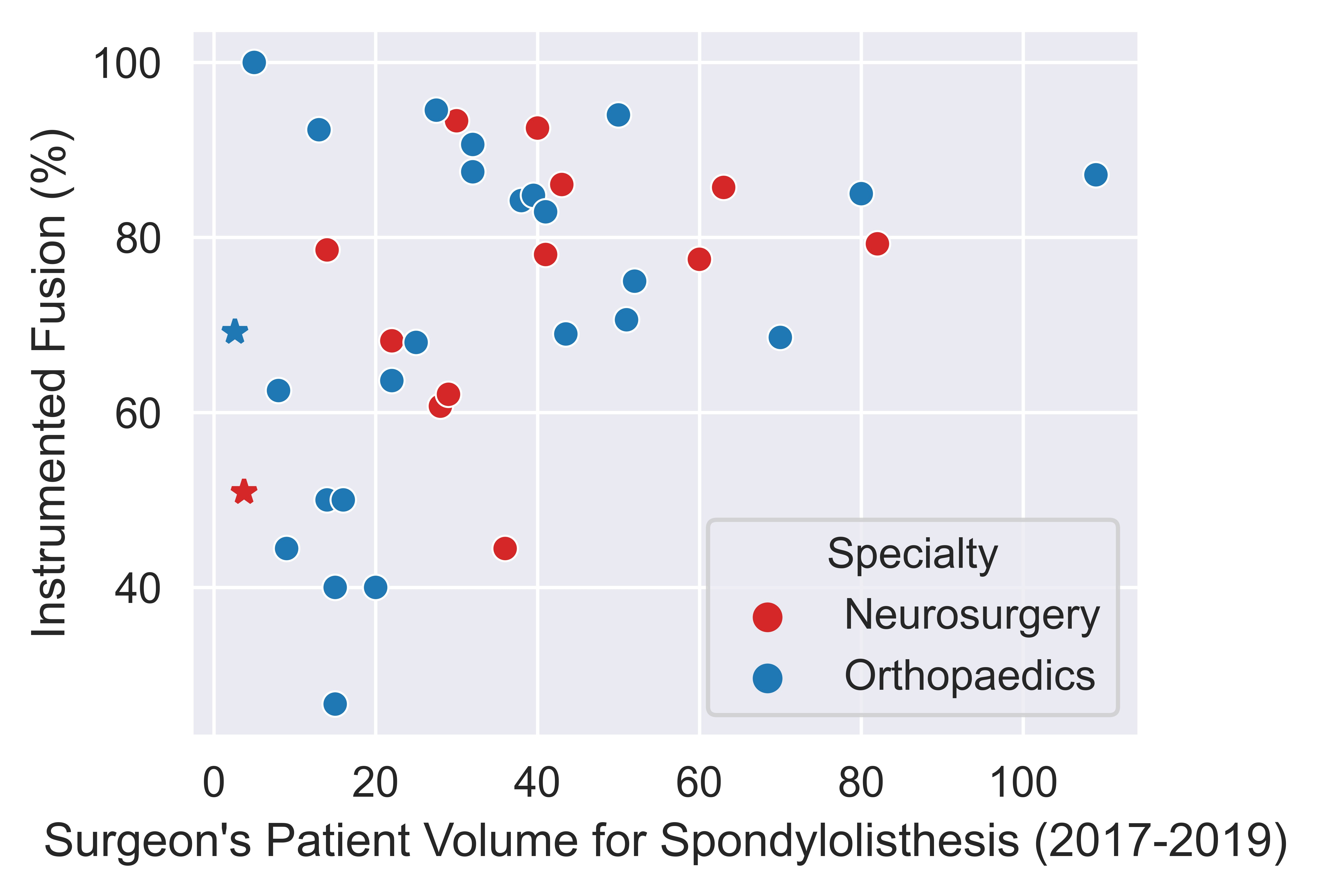 |

Your results are displayed with a red star if 4 or more relevant cases were identified for that diagnosis.

**Summary Table on Use of Instrumented Fusion Relative to Other Spine Surgeons:**

|  | **Rates Among All Surgeons by Percentile** | | | | | **Your Data** | |
| --- | --- | --- | --- | --- | --- | --- | --- |
| **Diagnosis** | **10th** | **25th** | **50th** | **75th** | **90th** | **Your Eligible Cases*** | **Your Rate*** |
| Spondylolisthesis | 0.0 | 0.4 | 0.69 | 0.88 | 1.0 | [N] | X.XX |
| Scoliosis | 0.0 | 0.08 | 0.44 | 0.63 | 0.85 | [N] | X.XX |

Note: Statistics for comparison surgeons represent all spine surgeons at both site 1 (N=42) and site 2 (N=47). *Eligible cases refers to the number of your surgical procedures for each diagnosis that were included in the study. Your rate was computed as the proportion of eligible cases that involved instrumented fusion (as defined by CPT codes).

**Surgical Appropriateness:**

| **Scoliosis** | | **Case 1** | **Case 2** | **Case 3** |
| --- | --- | --- | --- | --- |
| De-identified Patient MRN | | [MRN] | [MRN] | [MRN] |
| Was performing surgery appropriate? | | Yes |  |  |
| Was the procedure performed one of the most appropriate options? | | Yes |  |  |
| Procedure performed | | Decompression |  |  |
| Appropriateness Recommendations | |  |  |  |
|  | Decompression alone | Appropriate |  |  |
|  | Decompression and fusion | Appropriate |  |  |
|  | Decompression, fusion, and deformity correction | Uncertain |  |  |
|  | Fusion alone | Inappropriate |  |  |
|  | Fusion and deformity correction | Inappropriate |  |  |
| Clinical Characteristics | |  |  |  |
|  | Symptom severity | Severe |  |  |
|  | Stenosis severity (sx, signs, imaging) | Severe |  |  |
|  | # of levels with stenosis | 1-2 |  |  |
|  | Radiologic progression | No |  |  |
|  | Sagittal imbalance (signs, imaging) | Absent |  |  |
|  | Risk factors (psychol, clinical) | Moderate |  |  |
|  | Curvature | Curve 10-19 |  |  |

**Surgical Appropriateness:**

| **Spondylolisthesis** | | **Case 1** | **Case 2** | **Case 3** | **Case 4** |
| --- | --- | --- | --- | --- | --- |
| De-identified Patient MRN | | [MRN] | [MRN] |  |  |
| Was performing surgery appropriate? | | Intermediate | Yes |  |  |
| Was the procedure performed one of the most appropriate options? | | Yes | Yes |  |  |
| Procedure performed | | Decompression | Instrumented fusion |  |  |
| Appropriateness Recommendations | |  |  |  |  |
|  | Decompression alone | Uncertain | Inappropriate |  |  |
|  | Fusion without instrumentation (with/without decompression) | Uncertain | Uncertain |  |  |
|  | Fusion with instrumentation (with/without decompression) | Uncertain | Appropriate |  |  |
| Clinical Characteristics | |  |  |  |  |
|  | Main types of symptoms | Radicular pain | Only back pain |  |  |
|  | Back pain severity >=3 | Present | Present |  |  |
|  | Instability (sx, signs, imaging) | Absent | Present |  |  |
|  | Psychosocial risk factors | Present | Absent |  |  |
|  | Neurologic abnormality, severity | Severe | None |  |  |
|  | Type of significant stenosis (imaging) | Central and Foraminal | Significant foraminal stenosis |  |  |
|  | Medical comorbidities | Moderate | None/Mild |  |  |
|  | Disability | Severe | Severe |  |  |

**Suggestions for Improving Your Personal Performance:**

We will provide you with a new report card on your performance in 4 months.

You may want to consider the following strategies for improving your performance and alignment with the appropriate use criteria.

1. Ensure that you document key clinical characteristics needed to assess the risks and benefits of surgery for individual patients. We have created a “dot phrase” that auto populates an H&P template designed to make this easier.
2. Use free online calculators created by the AAOS to determine the appropriateness of each of the 5 categories of surgical procedures.
3. Scoliosis: [link]
4. Spondylolisthesis: [[link]](https://schulthess.webauthor.com/go/auc/)
5. Consider using the results from this calculator to support surgeon-patient communication and shared decision-making before surgery.
6. If you have used one of the online calculators and your professional judgement diverges from the calculator’s results, consider addend your last clinic note with a brief rationale with any additional information you may have considered. These comments can be taken into consideration when scoring appropriateness for future score cards.
7. Voluntarily self-refer your challenging clinical cases to our Multispecialty Case Conference. You may choose to do this, for example, if the patient requests a second opinion, if the appropriateness criteria indicate that operating would be “inappropriate” or recommends a different procedure than you were considering, or if you have other questions about the best approach for a case.
8. Attend the Multispecialty Case Conference as a participant, or serve on the Multispecialty Case Conference Core Committee, to learn in greater depth from a diversity of surgeon and non-surgeon colleagues.

**Refined Nudge Prototype: Description of Multispecialty Case Conference**

Purpose: More than just education, these conferences will engage surgeons as stakeholders and experts in the surgical appropriateness, explore the applicability of the criteria in specific clinical situations, identify potential refinements to keep the criteria up to date with evolving literature and standards of practice, and develop suggestions for navigating clinical situations where appropriateness is uncertain.

Invited Participants: All spine surgeons engaged in the ALIGN intervention will be invited. Attendance is optional.

Format and Timing: One-hour videoconferences will occur every other month at a convenient time. After the conference, surgeons will have access to video recordings and a dossier of conference proceedings.

Core Committee Members: Senior leaders in spine surgery at each site will invite respected experts in diverse specialties with experience in operative or non-operative approaches to degenerative spine conditions. Backgrounds may include: orthopaedic and neurosurgery-trained spine surgeons (including with and without fellowship training), physiatrists, pain management experts, geriatricians, primary care physicians, psychiatrists/ psychologists, and radiologists. On an annual basis, members would be rotated.

Preparations: To select cases for the conference, a Clinical Implementation Leader at each site would invite spine surgeons (participants) to self-refer cases for review and discussion, and also employ other diverse means (including chart reviews). Core Committee Members would review the suggested cases, add to them, and select 3-4 exemplar cases reflecting diverse scenarios for which surgery was “appropriate,” “uncertain,” or “rarely appropriate.” The Committee Members would also suggest any key literature (articles, guidelines, other resources) to consider for each case, drawing from a regularly updated search. For each exemplar case, the Clinical Implementation Leader would then score the appropriateness criteria and develop slides for the meeting that describe symptoms, signs, psychosocial factors, clinical comorbidities, and imaging as well as appropriateness criteria recommendations and brief summaries of relevant literature.

Meeting Proceedings: Each conference would discuss 3-4 cases. The Clinical Implementation Lead would present the slides and invite the participants to share their opinions about whether the patient is a good candidate for surgery and the pros and cons of the alternative operative approaches. Core Committee Members would reflect on the clinical case, discussion, and literature, and then formulate suggestions for surgeons to consider in similar situations in the future. A quick vote among Core Committee members will lead to endorsements of each suggestion, by majority opinion. The case, associated appropriateness recommendations, relevant literature, and endorsed committee suggestions will be recorded in an online dossier. This dossier will be available to surgeons whether or not they attended the conference.

Resources during and after the Conference:

- Dossiers created through conference deliberations
- Online appropriateness calculators:
  - Scoliosis Tool: [link]
  - Spondylolisthesis Tool: [link]
  - Supporting materials: [link]

Evidence Base for Conference Approach: Multispecialty case conferences, sometimes called tumor boards in cancer care or multidisciplinary team meetings in other contexts, have been widely implemented in care delivery. For cancer care, previous research indicates that these conferences improve appropriate treatment selection, while the literature on their effectiveness is limited. Multispecialty case conferences, as a component of a comprehensive initiative to improve adult scoliosis surgery outcomes, have been shown to reduce complications. Implementation studies have identified key characteristics to promote conference use, including provider and administrator consensus on usefulness and buy-in to integrating conferences into clinical workflows.

**Refined Nudge Prototype: Preop Check Sample Email**

***Subject Line:*** Please Review Preop Check for Surgical Patient

***Body of email***

Dear Dr. [Name],

Please review the appropriateness recommendations below for your planned operation. If the recommendations differ from what you might have been considering, please:

- Explore the recommendations further using an **online appropriateness calculator**:
  - Scoliosis Tool: [link]
  - Spondylolisthesis Tool: [link]
  - Supporting materials: [link]
- **Addend your last visit note** with information that might have influenced your procedure selection.
- Join our **bimonthly multispecialty case conference**, where spine surgeons and other experts in spine conditions debate appropriate surgical options for diverse and often challenging clinical situations.
- **Share your feedback:** email our Clinical Implementation Leader, [name], at [email].

Sincerely,

[Site leader name]

**Patient:** [Name] **MRN:** [MRN]

**Diagnosis:** [Scoliosis and/or spondylolisthesis] **OR Date:** [Planned date of surgery]

[Note: Below is an original drawing by the study investigators that depicts the resulting information.]


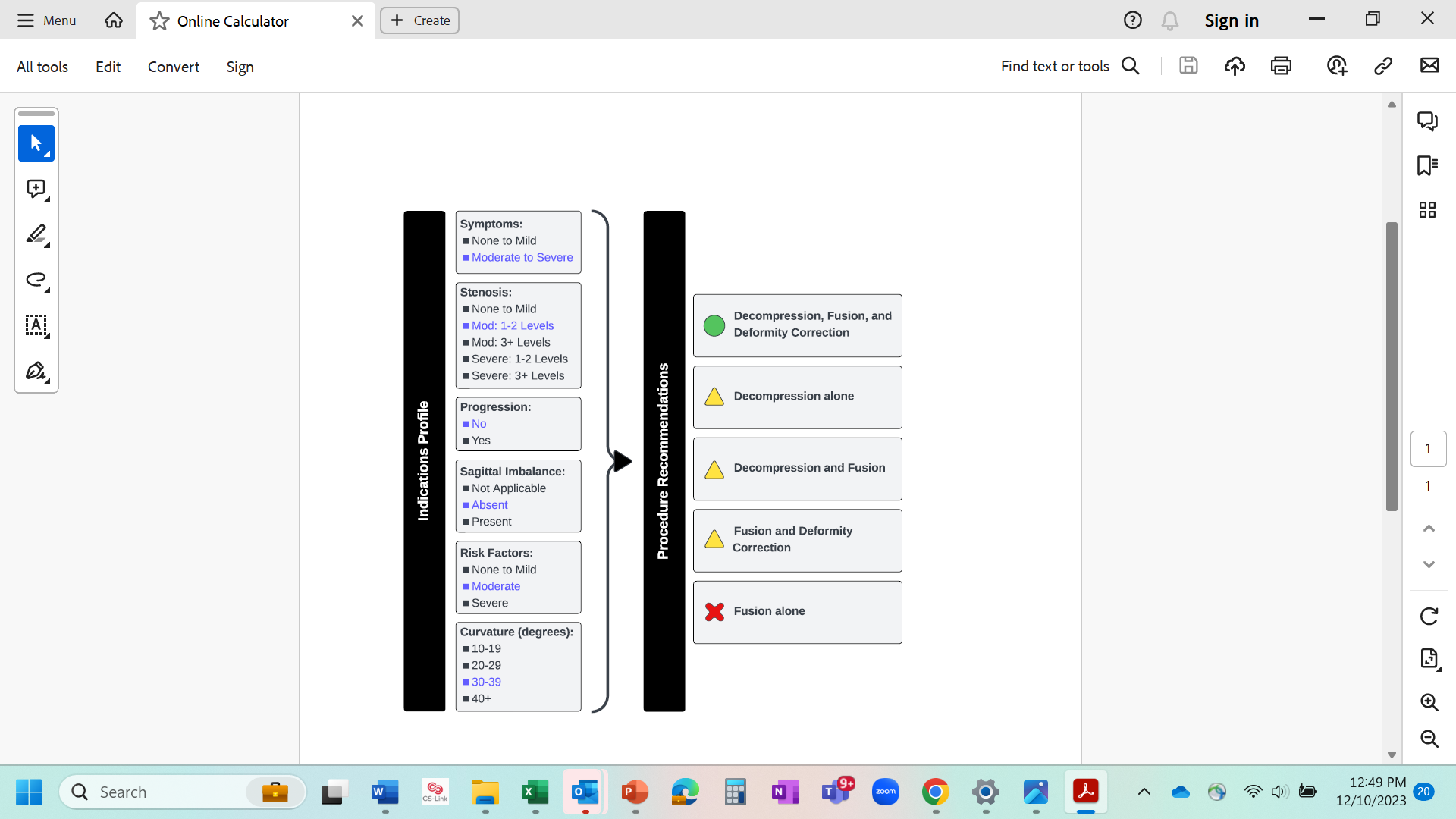


**Pilot Testing of Refined Nudge Prototypes: Mean Responses and Standard Deviation (SD), N=5 Spine Surgeons**

| ***Nudge Prototype and Associated Survey Items* (1-5 scale where 5=strongly agree)** | | ***Mean (SD)*** |
| --- | --- | --- |
| **Preoperative Appropriateness Check** | |  |
| *Acceptability:* | |  |
|  | 1. I would approve of use the preoperative appropriateness check in my practice | 4.0 (0.7) |
|  | 1. I would feel comfortable receiving this preoperative appropriateness check | 4.2 (0.4) |
|  | 1. This preoperative appropriateness check would help me feel more supported in my decision-making | 3.8 (0.8) |
| *Applicability:* | |  |
|  | 1. This preoperative appropriateness check is applicable to my practice | 3.8 (0.4) |
|  | 1. This preoperative appropriateness check would be helpful for me | 3.8 (0.4) |
|  | 1. This preoperative appropriateness check would be helpful for other spine surgeons | 3.8 (0.4) |
| *Feasibility:* | |  |
|  | 1. This preoperative appropriateness check seems like it could be implemented in surgical practice | 3.8 (0.4) |
|  | 1. The mockup personalized preoperative appropriateness checks seemed like they would be easy for me to use | 4.0 (0.0) |
| **Online Appropriateness Calculators** | |  |
| *Acceptability:* | |  |
|  | 1. I would approve of use these appropriateness calculators in my practice | 3.8 (0.4) |
|  | 1. I would feel comfortable receiving recommendations from these appropriateness calculators | 4.0 (0.0) |
|  | 1. These appropriateness calculators would help me feel more supported in my decision-making | 3.8 (0.4) |
| *Applicability:* | |  |
|  | 1. These appropriateness calculators are applicable to my practice | 3.8 (0.4) |
|  | 1. These appropriateness calculators would be helpful for me | 3.8 (0.4) |
|  | 1. These appropriateness calculators would be helpful for other spine surgeons | 3.8 (0.4) |
| *Feasibility:* | |  |
|  | 1. These appropriateness calculators seem like they could be implemented in surgical practice | 3.8 (0.4) |
|  | 1. These appropriateness calculators were easy for me to use | 4.0 (0.0) |
| **Individualized Surgeon Score card** | |  |
| *Acceptability:* | |  |
|  | 1. I would approve of use this score card in my practice | 4.0 (0.0) |
|  | 1. I would feel comfortable receiving this score card | 4.2 (0.4) |
|  | 1. This score card would help me feel more supported in my decision-making | 3.8 (0.4) |
| *Applicability:* | |  |
|  | 1. This score card is applicable to my practice | 3.8 (0.4) |
|  | 1. This score card would be helpful for me | 3.8 (0.4) |
|  | 1. This score card would be helpful for other spine surgeons | 3.8 (0.4) |
| *Feasibility:* | |  |
|  | 1. This score card seems like it could be implemented in surgical practice | 3.8 (0.4) |
|  | 1. The score card was easy for me to use | 3.8 (0.4) |
| **Multispecialty Case Conference** | |  |
| *Acceptability:* | |  |
|  | 1. I would approve of use the case conferences described above in my practice | 4.4 (0.5) |
|  | 1. I would feel comfortable participating in the case conferences described above | 4.4 (0.5) |
|  | 1. The case conferences described above would help me feel more supported in my decision-making | 4.4 (0.5) |
| *Applicability:* | |  |
|  | 1. The case conferences described above is applicable to my practice | 4.4 (0.5) |
|  | 1. The case conferences described above would be helpful for me | 4.4 (0.5) |
|  | 1. The case conferences described above would be helpful for other spine surgeons | 4.2 (0.8) |
| *Feasibility:* | |  |
|  | 1. The case conferences described above seems like it could be implemented in surgical practice | 4.4 (0.7) |
|  | 1. The case conferences described above seemed like they would be easy for me to use | 4.4 (0.5) |
