# Supplementary material for: Surgical appropriateness nudges: Developing behavioral science nudges to integrate appropriateness criteria into the decision making of spine surgeons
Source: PLoS One. 2024 Apr 19;19(4):e0300475. doi: 10.1371/journal.pone.0300475 (PMC11029649; doi:10.1371/journal.pone.0300475)
Supplement: S2 File — (DOCX) [file pone.0300475.s002.docx]

**S2 File: Map of Surgical Workflow**

**“Maps” of Preoperative Workflows at Two Regional Referral Centers for Spine Surgery**

| **Institution** | **Referral 🡪** | **Surgeon Assess Risks/Benefits 🡪** | **Surgeon Discusses with Patient 🡪** | **Quality Assurance Procedures 🡪** | **Intra-operative Decision-making** |
| --- | --- | --- | --- | --- | --- |
| **Common to both institutions** | • Studies (MRI, X-ray) usually completed in advance • Prior conservative management failed | • Good history & physical critical  • Elicit patient goals  • Order additional studies as needed  • Nearly all decision-making about whether to offer surgery (and which procedure) occurs at this stage  • Data for applying AUC not available in electronic format | • Patients weigh options • Patients seek second opinion if desired | • Patient scheduled for surgery after agreement by patient and surgeon • No formal review by department or hospital • Informal case review sought from colleagues | • Typically, no major changes to procedure choice or approach immediately before or during procedure • No formal feedback mechanism outside of mortality and morbidity review |
| **Site 1 only** | • Referral accepted from all sources, including primary care | • Process for seeing patients not standardized between surgeons |  | • Educational conferences discuss cases with residents and fellows (ad hoc) |  |
| **Site 2 only** | • Referrals accepted from specialists (neurosurgery, orthopedics, physical medicine and rehabilitation, pain management, neurology, or specialized spine clinic) | • Surgeons given up to 1 hour for new consults  • Surgeons are given 20 minutes for visits for second opinions  • Order additional studies as needed but most studies are readily available through electronic health record  • Patients generally receive three options, although some patients with more co-morbidities may only receive less-invasive options | • Patient given 2 weeks to weigh options • Second opinion often sought when surgery not offered or when recommended procedure more invasive than desired by patient | • New technologies are assessed by central Medical Technology Assessment Team (MTAT) team • Surgeons may seek assistance on complex cases |  |
