# Supplementary material for: Surgical appropriateness nudges: Developing behavioral science nudges to integrate appropriateness criteria into the decision making of spine surgeons
Source: PLoS One. 2024 Apr 19;19(4):e0300475. doi: 10.1371/journal.pone.0300475 (PMC11029649; doi:10.1371/journal.pone.0300475)
Supplement: S3 File — (DOCX) [file pone.0300475.s003.docx]

**S3 File: Focus Group 2 Results**

| **Nudge Type** | **Surgeon responses** | **Illustrative quotes** |
| --- | --- | --- |
| Structured note template | If it helps facilitate efficiency and completeness of documentation, it would garner high levels of buy-in  In systems with open notes, patients may review their providers’ clinical notes. The structured note template could help patients have more confidence in the ultimate surgical decision  Ease of use is vital.  Documentation practices can be highly variable which might make structured note template challenging. Some surgeons rely on spine fellows, rotating residents, and/or physicians assistants to document clinic notes; other surgeons use dictation or voice recognition software to generate clinic notes. | “Any approach for minimizing work would get buy-in quickly. For instance, an EPIC template that could pull in data quickly and captures data surgeons need to have anyway.”  “[While it’s] important to adopt standardized dot phrases to streamline workflow… [it] has to be easy to use and flexible because surgical decision-making is nuanced.” |
| Online calculator | Surgeons already bookmark online calculators for other conditions and use them frequently when the clinical situation is appropriate.  Existing calculators could be augmented to be even more useful to surgeons. | “A phrase that would pop up after using the calculator online would be useful because surgeons could paste the phrase into their note. Something like ‘I used the AAOS DLS Appropriateness Calculator which rated patients with this clinical scenario as appropriate for surgical intervention.’ with a reference, could help justify surgeon’s decision making and be pasted into the clinical note.” |
| Multispecialty conferences | Surgeons noted they really need additional support in scenarios for which the appropriateness method does not make a recommendation on appropriateness or inappropriateness. Multispecialty conferences are already fairly common in surgical training.  Multispecialty conference should be optional and not required.  Important to set ground rules so that all participants have equal opportunities to contribute. | “The presence of a few strong personalities might stifle open discussion.”  Without careful planning, such a conference could also “homogenize to the institution’s culture” (e.g. result in regression to the mean).  Group size for such conference is an important consideration since groups that are too large often mean that “decisions don’t get made”. |
| Accountable justification | Surgeons receptive to accountable justification, but did not have strong feelings or discuss this type of nudge in great detail. | “It could be useful, it’s nice to have a flag for providing the rationale for a procedure.”  Accountable justification could “help to track behavior”. |
| Individualized score card | **Individualized score card** received mixed reviews from surgeons.  If the score card is used for public shaming, it could be problematic.  Concerns that the score cards could be used punitively (e.g., to decrease operating room time).  Scoring approach must be transparent and logical. Patient case identification should be shared because ICD-10 codes may not always be accurately applied.  Several surgeons were very interested to see how their practices and outcomes compared with peers, particularly their closest peers within their own practice groups. | “Maybe high performer public reporting would be good, but individual results should not be publicly shared.” |
