# Supplementary material for: Surgical appropriateness nudges: Developing behavioral science nudges to integrate appropriateness criteria into the decision making of spine surgeons
Source: PLoS One. 2024 Apr 19;19(4):e0300475. doi: 10.1371/journal.pone.0300475 (PMC11029649; doi:10.1371/journal.pone.0300475)
Supplement: S4 File — (DOCX) [file pone.0300475.s004.docx]

**S4 File: Nudge Frameworks Considered, Inclusion/Exclusion, and Rationale** (citations 21, 22, 60, 61, 62, 63, 64, 65)

| Nudge Framework | Example from Setting in Healthcare | Inclusion/Exclusion in Refined Nudge Prototypes | Rationale |
| --- | --- | --- | --- |
| **Nudge Frameworks Included in Preliminary Nudge Prototypes** | | | |
| **Peer comparisons:** Providing individuals with data on their performance relative to similar peers; references descriptive norms | Comparing physician antibiotic prescribing rates with those of “top performers.” (Meeker et al., 2016; Linder et al., 2017) | *Yes, included in Individualized Score Cards* | Surgeons value but do not consistently receive information about their performance. They value most highly data on peers within their own practices. |
| **Descriptive norm:** Reflects how people actually behave in practice. | Same as above | *Yes, included in Individualized Score Cards* | Same as above |
| **Feedback:** Reporting the frequency or occurrence of the target behavior back to the user | Same as above | *Yes, included in Individualized Score Cards* | Some surgeons are accustomed to score cards and would appreciate additional feedback to support improvement. |
| **Framing:** People pay more attention to negative vs. positive information | Physicians prefer a riskier treatment when outcomes are presented as lives lost rather than lives saved (Almashat et al. 2008) | *Relevant to Individualized Score Cards, but ultimately excluded* | Feedback to clinicians should be presented in non-judgmental, constructive way to avoid triggering defensive reactions. |
| **Decision Aid:** Tools that help with considering several different options | Tools to help with deciding about stool testing in colorectal cancer screening (Schwartz et al. 2017) | *Yes, included in Online Calculator* | Surgeons felt appropriateness calculators could complement many other nudge frameworks as well as be used on their own. |
| **Mapping:** Simplifying information about the relationship between choice alternatives and the resulting  outcomes | Emphasizing implications of hand hygiene for patients hygiene (Grant and Hofmann, 2011) | *Yes, included in Online Calculator* | Employing simple symbols and colors draws attention to the anticipated risks vs. benefits of surgical options. |
| **Injunctive Social Norm:** Presenting a desired decision as common and “normal” behavior | Health care team leaders led activities in their units to encourage hand hygiene (Huis et al., 2012) | *Yes, included in Multispecialty Case Conference* | Surgeons value the opinions of thought leaders in their fields and want to “do well”. |
| **Reminders/Alerts:** Calling people’s attention to a desired behavior with timely message | Hospital staff received reminders/alerts for annual influenza vaccination (Schmidtke et al. 2019) | *Yes, included in Preoperative Check* | Surgeons appreciated the additional support to being able to provide explanation for why certain aspects of surgical decision-making may not fall within strictly defined criteria. |
| **Salience of Information:** Presenting information relevant to a decision in a way that enhances its prominence or visibility | Present information on costs to physicians at the time of test ordering to avoid unnecessary testing (Sedrak et al., 2017) | *Yes, included in Preoperative Check* | Providing information tailored to individual patients before surgery may have the greatest potential to shape surgeon behavior. |
| **Accountable justification:** Asking individuals to document rationale for making less desired decisions. | Electronically prompting clinicians to document justifications for potentially inappropriate antibiotics (Meeker et al., 2016) | *Yes, included in Preoperative Check* | Surgeons welcomed an opportunity to explain why certain aspects of surgical decision-making do not fall within strictly defined criteria. |
| **Defaults:** A particular choice is ‘preset’, making it the easiest option | Setting prescription drugs to default to generic equivalents (Malhotra et al., 2016) | *Relates to the Structured Note Template discussed in Focus Group 2, but ultimately excluded* | Documentation practices are too highly variable to rely on a structured note template. Recommended care varies greatly based on patient characteristics, and this makes other applications of defaults risky. |
| **Common Nudge Frameworks Excluded from Preliminary Nudge Prototypes:** These nudges were challenging to apply to complex decision making where there are multiple common surgical procedures that can be used, and recommended care differs greatly across patients. | | | |
| **Priming:** Create physical, verbal or sensational cues that subconsciously shift participants toward a particular choice | Placing hand sanitizer dispenser to improve hand hygiene among anesthesiology staff in the operating room (Munoz-Price et al. 2014) | *No, excluded* | Choosing a surgical procedure for an individual patient is an active not subconscious choice. |
| **Menu partition:** Splitting potential options into groups to facilitate selection of a recommended option | Listing narrow-spectrum antibiotics individually while grouping broad-spectrum antibiotics together (Tannenbaum et al., 2015) | *No, excluded* | Surgeons’ notes are often not completed until after the patient visit, creating mismatch with the need to present surgical options in real-time. |
| **Anchors:** Initial exposure to a number serves as a reference point and influences decisions | E-prescribing platforms automatically set new opioid prescriptions to 10 tablets (Delgado et al., 2018) | *No, excluded* | Numbers are not relevant to the selection of a surgical procedure. |
| **Pre-commitment:** Asking individuals to commit to a decision in advance of actually making the decision | Asking physicians to post exam room signs committing to appropriate antibiotic prescribing (Meeker et al., 2014) | *No, excluded* | Framework assumes the decision is straightforward and easy to identify and adhere to. Surgical decision making is more complex and subject to interpretation so it’s hard for surgeons to meaningfully make a pre-commitment. |
