# Supplementary material for: Surgical appropriateness nudges: Developing behavioral science nudges to integrate appropriateness criteria into the decision making of spine surgeons
Source: PLoS One. 2024 Apr 19;19(4):e0300475. doi: 10.1371/journal.pone.0300475 (PMC11029649; doi:10.1371/journal.pone.0300475)

**S5 File: Refined Nudge Prototypes:** **Details on Nudge Design, Examples**

Online Calculators: See text and figure in text for description of the calculators and example of an application to a hypothetical patient.

Individualized Surgeon Score cards: We developed a model score card that presented data on each surgeon’s use of instrumented fusion and rates of major in-hospital complications relative to peers across both study sites, as well as adherence to appropriateness criteria.

First, we created tables and graphics with rates of instrumented fusion and major in-hospital surgical complications for the individual participating surgeon and 88 eligible peers. This involved specifying ICD-10-CM and CPT codes, obtaining administrative data for 2017-2019, identifying 89 eligible surgeons and 2,481 eligible operations, and calculating surgeon-level rates of instrumented fusion as well as major in-hospital complications (hemorrhage, mechanical complications, dural tears, surgical site infections, anesthesia complications, or mortality during the index hospitalization).

Second, the model score cards illustrated how adherence to appropriateness criteria would be reported. For the six participating surgeons and 6 additional surgeons (3 per site) chosen at random, we randomly selected five patients each (60 total). We then trained nurses and physician assistants at study sites to manually review of the selected patients’ medical records, apply additional eligibility criteria specific to the appropriateness criteria, and score adherence to the appropriateness criteria. This produced data on adherence for 2-4 patients per surgeon.

See sample score card for hypothetical spine surgeon below.

In the pilot tests, we created a score card and distributed it to each of the 6 participating surgeons.

Multispecialty Case Conference: See below.

In the pilot tests, we shared this description with the six participating surgeons.

Preoperative Appropriateness Checks (“Preop Checks”): Preop checks would involve using the EHR to detect when surgeons schedule patients for eligible operations, and then emailing the surgeon a set of recommendations based on the appropriateness criteria as well as relevant resources for assessing appropriateness themselves. Each email would outline the patient’s clinical characteristics related to appropriateness, the appropriateness recommendation for the patient, and actions the surgeons could take if the recommendations diverged from the operation they had planned. The patients and data included in the preop checks were the same as those included in the score cards.

See sample preop check for a hypothetical spine surgeon below.

In the pilot tests, we sent two sample preop check emails to each of the six participating surgeons (12 patients total). The patients for whom we presented data were the same as for the individualized score card.

**Refined Nudge Prototype: Individualized Score Card for Hypothetical Surgeon**

**Surgical Outcomes and Volume:**

**Period of Time Covered:** January 1, 2017 – December 31, 2019

Number of Operations Reviewed: [N]*

**Part 1: Your Major In-hospital Complication Rate Relative to Other Spine Surgeons:**

| Scoliosis | Spondylolisthesis |
| --- | --- |
| 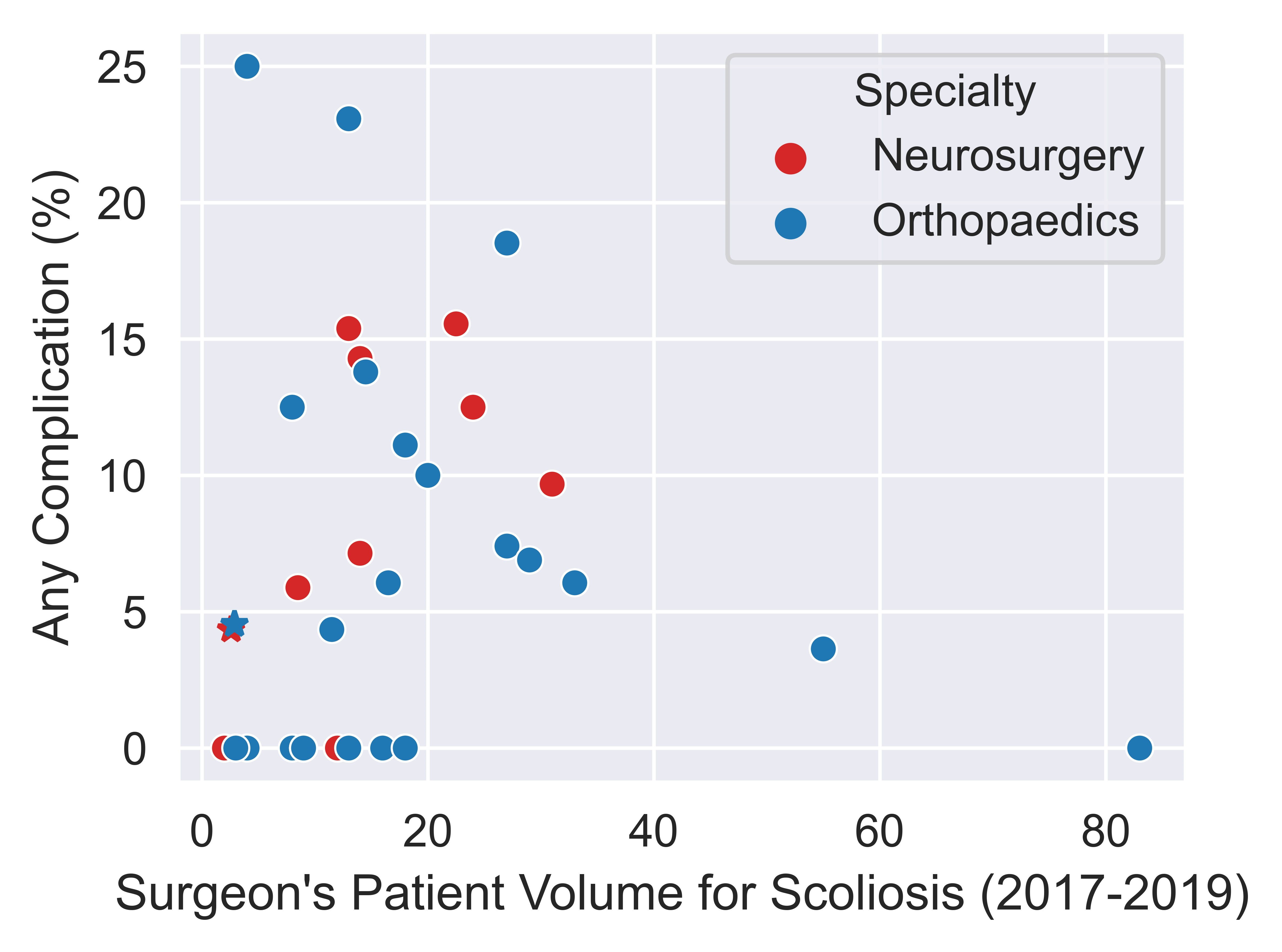 | 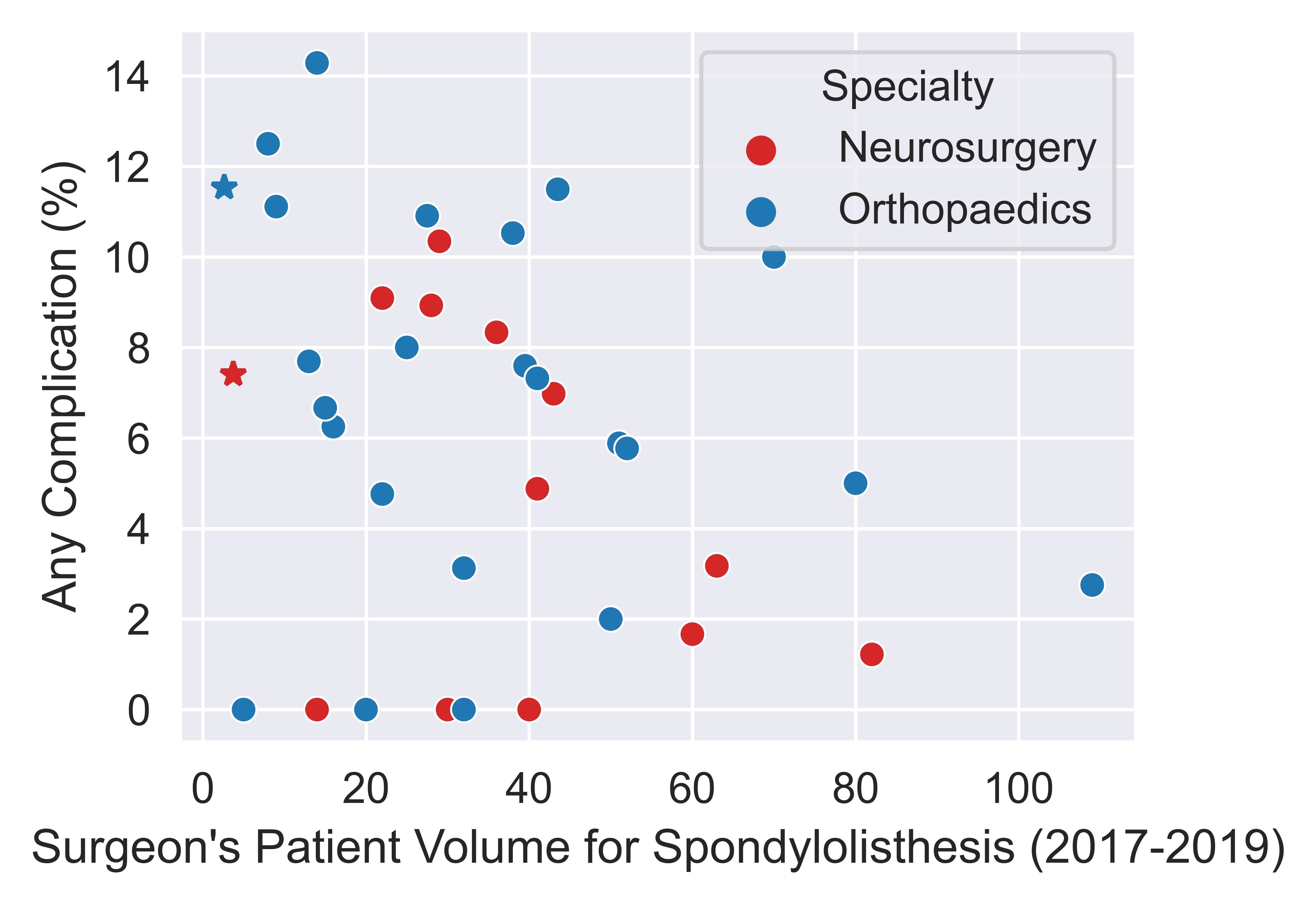 |

Your results are displayed with a red star if 4 or more relevant cases were identified for that diagnosis.

**Summary Table on Complications Relative to Other Spine Surgeons:**

|  | **Rates Among All Surgeons by Percentile** | | | | | **Your Data** | |
| --- | --- | --- | --- | --- | --- | --- | --- |
| **Diagnosis** | **10th** | **25th** | **50th** | **75th** | **90th** | **Your Eligible Cases*** | **Your Rate*** |
| Spondylolisthesis | 0.0 | 0.0 | 0.05 | 0.1 | 0.21 | [N] | X.XX |
| Scoliosis | 0.0 | 0.0 | 0.0 | 0.07 | 0.17 | [N] | X.XX |

Note: Statistics for comparison surgeons represent all spine surgeons at both site 1 (N=42) and site 2 (N=47). *Eligible cases refers to the number of your surgical procedures for each diagnosis that were included in the study. Your rate was computed as the proportion of eligible cases that recorded one or more surgical complications (as defined by ICD-10 codes).

**Specific patients of yours who were identified as having inpatient complications are listed below:**

| **Deidentified Patient MRN** | **Diagnosis** | **Instrumented Fusion** | **Complication(s)** |
| --- | --- | --- | --- |
| [XXXXXXXXXXX] | Spondylolisthesis | Yes | [From list below] |
| [XXXXXXXXXXX] | Scoliosis | Yes | [From list below] |
| [XXXXXXXXXXX] | Scoliosis | No | [From list below] |

* All surgical cases were identified from electronic health record system at your institution based on International Classification of Diseases, 10^th^ edition (ICD-10) and Current Procedural Terminology (CPT) codes. Complications were identified using ICD-10 codes. Major in-hospital complications included: hemorrhage, mechanical complications, dural tears, surgical site infections, anesthesia complications, or mortality during the index hospitalization.

**Part 2: Your Use of Instrumented Fusion Relative to Other Spine Surgeons:**

| Scoliosis | Spondylolisthesis |
| --- | --- |
| 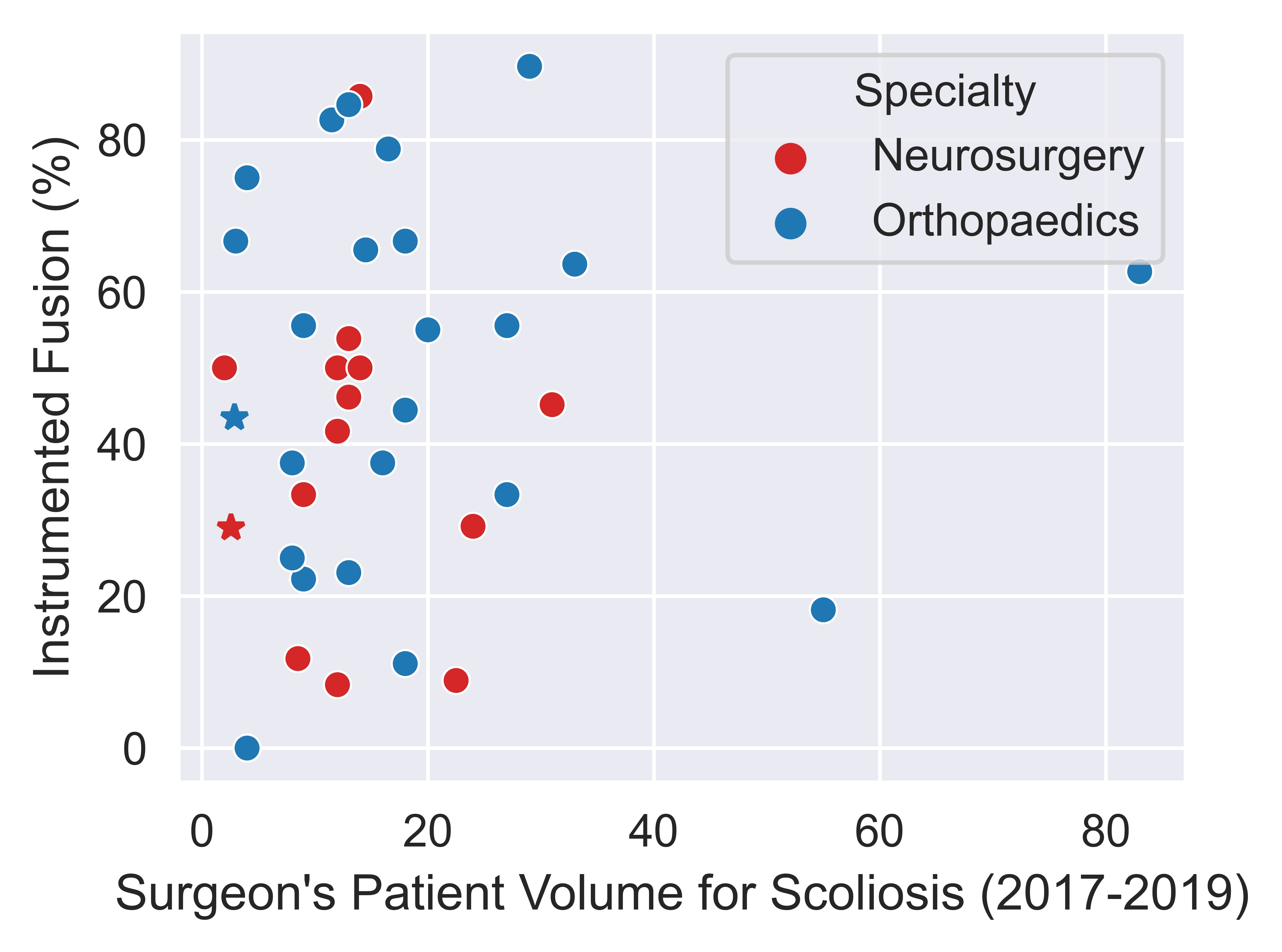 | 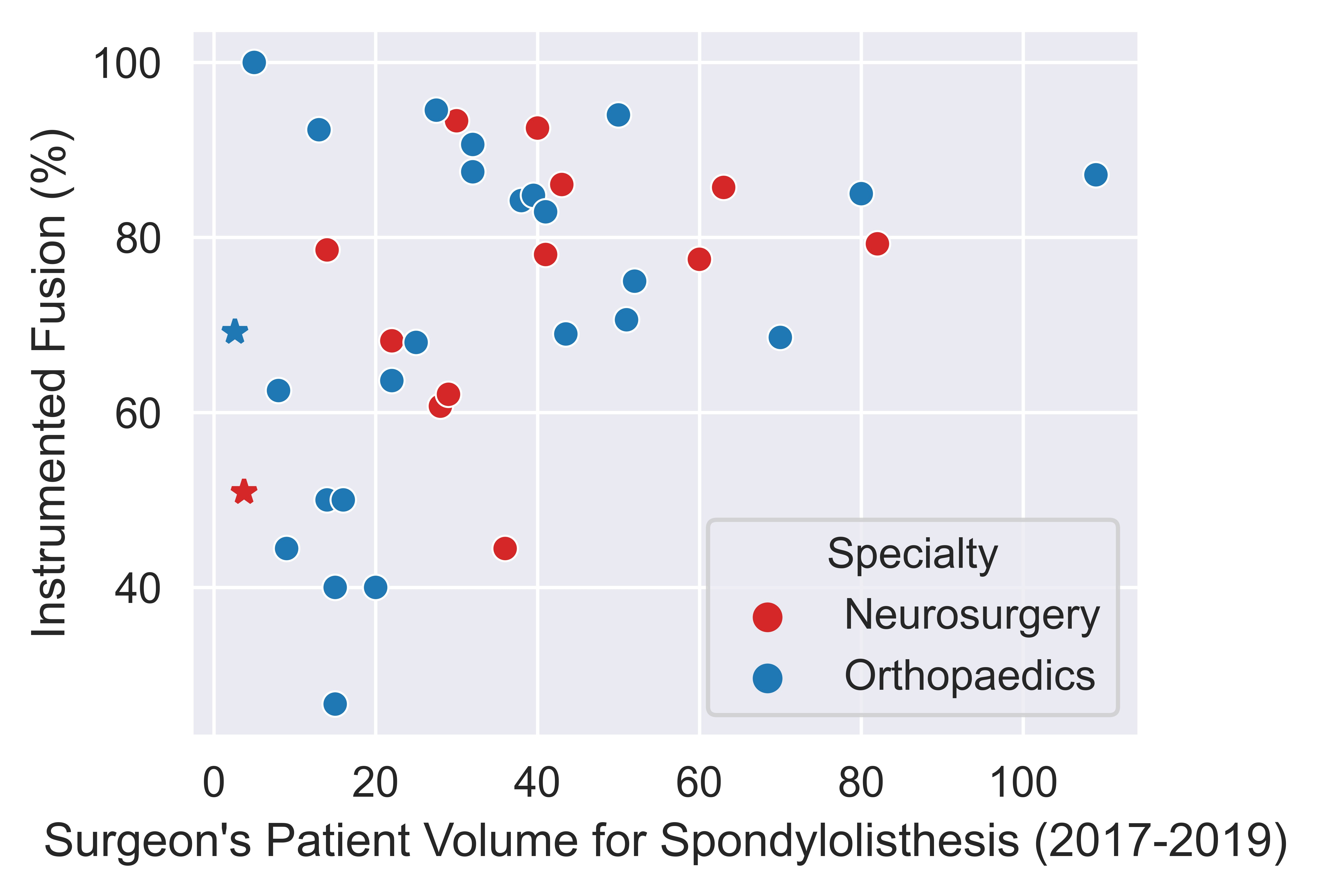 |

Your results are displayed with a red star if 4 or more relevant cases were identified for that diagnosis.

**Summary Table on Use of Instrumented Fusion Relative to Other Spine Surgeons:**

|  | **Rates Among All Surgeons by Percentile** | | | | | **Your Data** | |
| --- | --- | --- | --- | --- | --- | --- | --- |
| **Diagnosis** | **10th** | **25th** | **50th** | **75th** | **90th** | **Your Eligible Cases*** | **Your Rate*** |
| Spondylolisthesis | 0.0 | 0.4 | 0.69 | 0.88 | 1.0 | [N] | X.XX |
| Scoliosis | 0.0 | 0.08 | 0.44 | 0.63 | 0.85 | [N] | X.XX |

Note: Statistics for comparison surgeons represent all spine surgeons at both site 1 (N=42) and site 2 (N=47). *Eligible cases refers to the number of your surgical procedures for each diagnosis that were included in the study. Your rate was computed as the proportion of eligible cases that involved instrumented fusion (as defined by CPT codes)

**Surgical Appropriateness:**

| **Scoliosis** | | **Case 1** | **Case 2** | **Case 3** |
| --- | --- | --- | --- | --- |
| De-identified Patient MRN | | [MRN] | [MRN] | [MRN] |
| Was performing surgery appropriate? | | Yes |  |  |
| Was the procedure performed one of the most appropriate options? | | Yes |  |  |
| Procedure performed | | Decompression |  |  |
| Appropriateness Recommendations | |  |  |  |
|  | Decompression alone | Appropriate |  |  |
|  | Decompression and fusion | Appropriate |  |  |
|  | Decompression, fusion, and deformity correction | Uncertain |  |  |
|  | Fusion alone | Inappropriate |  |  |
|  | Fusion and deformity correction | Inappropriate |  |  |
| Clinical Characteristics | |  |  |  |
|  | Symptom severity | Severe |  |  |
|  | Stenosis severity (sx, signs, imaging) | Severe |  |  |
|  | # of levels with stenosis | 1-2 |  |  |
|  | Radiologic progression | No |  |  |
|  | Sagittal imbalance (signs, imaging) | Absent |  |  |
|  | Risk factors (psychol, clinical) | Moderate |  |  |
|  | Curvature | Curve 10-19 |  |  |

| **Spondylolisthesis** | | **Case 1** | **Case 2** | **Case 3** | **Case 4** |
| --- | --- | --- | --- | --- | --- |
| De-identified Patient MRN | | [MRN] | [MRN] |  |  |
| Was performing surgery appropriate? | | Intermediate | Yes |  |  |
| Was the procedure performed one of the most appropriate options? | | Yes | Yes |  |  |
| Procedure performed | | Decompression | Instrumented fusion |  |  |
| Appropriateness Recommendations | |  |  |  |  |
|  | Decompression alone | Uncertain | Inappropriate |  |  |
|  | Fusion without instrumentation (with/without decompression) | Uncertain | Uncertain |  |  |
|  | Fusion with instrumentation (with/without decompression) | Uncertain | Appropriate |  |  |
| Clinical Characteristics | |  |  |  |  |
|  | Main types of symptoms | Radicular pain | Only back pain |  |  |
|  | Back pain severity >=3 | Present | Present |  |  |
|  | Instability (sx, signs, imaging) | Absent | Present |  |  |
|  | Psychosocial risk factors | Present | Absent |  |  |
|  | Neurologic abnormality, severity | Severe | None |  |  |
|  | Type of significant stenosis (imaging) | Central and Foraminal | Significant foraminal stenosis |  |  |
|  | Medical comorbidities | Moderate | None/Mild |  |  |
|  | Disability | Severe | Severe |  |  |

**Suggestions for Improving Your Personal Performance:**

We will provide you with a new report card on your performance in 4 months.

You may want to consider the following strategies for improving your performance and alignment with the appropriate use criteria.

1. Ensure that you document key clinical characteristics needed to assess the risks and benefits of surgery for individual patients. We have created a “dot phrase” that auto populates an H&P template designed to make this easier.
2. Use free online calculators created by the AAOS to determine the appropriateness of each of the 5 categories of surgical procedures.
3. Scoliosis: [link]
4. Spondylolisthesis: [[link]](https://schulthess.webauthor.com/go/auc/)
5. Consider using the results from this calculator to support surgeon-patient communication and shared decision-making before surgery.
6. If you have used one of the online calculators and your professional judgement diverges from the calculator’s results, consider addend your last clinic note with a brief rationale with any additional information you may have considered. These comments can be taken into consideration when scoring appropriateness for future score cards.
7. Voluntarily self-refer your challenging clinical cases to our Multispecialty Case Conference. You may choose to do this, for example, if the patient requests a second opinion, if the appropriateness criteria indicate that operating would be “inappropriate” or recommends a different procedure than you were considering, or if you have other questions about the best approach for a case.
8. Attend the Multispecialty Case Conference as a participant, or serve on the Multispecialty Case Conference Core Committee, to learn in greater depth from a diversity of surgeon and non-surgeon colleagues.

**Refined Nudge Prototype: Multispecialty Case Conference**

Purpose: More than just education, these conferences will engage surgeons as stakeholders and experts in the surgical appropriateness, explore the applicability of the criteria in specific clinical situations, identify potential refinements to keep the criteria up to date with evolving literature and standards of practice, and develop suggestions for navigating clinical situations where appropriateness is uncertain.

Participants: All spine surgeons engaged in the ALIGN intervention will be invited. Attendance is optional.

Format and Timing: One-hour videoconferences will occur every other month at a convenient time. After the conference, surgeons will have access to video recordings and a dossier of conference proceedings.

Core Committee Members: Senior leaders in spine surgery at each site will invite respected experts in diverse specialties with experience in operative or non-operative approaches to degenerative spine conditions. Backgrounds may include: orthopaedic and neurosurgery-trained spine surgeons (including with and without fellowship training), physiatrists, pain management experts, geriatricians, primary care physicians, psychiatrists/ psychologists, and radiologists. On an annual basis, members would be rotated.

Preparations: To select cases for the conference, a Clinical Implementation Leader at each site would invite spine surgeons (participants) to self-refer cases for review and discussion, and also employ other diverse means (including chart reviews). Core Committee Members would review the suggested cases, add to them, and select 3-4 exemplar cases reflecting diverse scenarios for which surgery was “appropriate,” “uncertain,” or “rarely appropriate.” The Committee Members would also suggest any key literature (articles, guidelines, other resources) to consider for each case, drawing from a regularly updated search. For each exemplar case, the Clinical Implementation Leader would then score the appropriateness criteria and develop slides for the meeting that describe symptoms, signs, psychosocial factors, clinical comorbidities, and imaging as well as appropriateness criteria recommendations and brief summaries of relevant literature.

Meeting Proceedings: Each conference would discuss 3-4 cases. The Clinical Implementation Lead would present the slides and invite the participants to share their opinions about whether the patient is a good candidate for surgery and the pros and cons of the alternative operative approaches. Core Committee Members would reflect on the clinical case, discussion, and literature, and then formulate suggestions for surgeons to consider in similar situations in the future. A quick vote among Core Committee members will lead to endorsements of each suggestion, by majority opinion. The case, associated appropriateness recommendations, relevant literature, and endorsed committee suggestions will be recorded in an online dossier. This dossier will be available to surgeons whether or not they attended the conference.

Resources during and after the Conference:

- Dossiers created through conference deliberations
- Online appropriateness calculators:
  - Scoliosis Tool: [link]
  - Spondylolisthesis Tool: [link]
  - Supporting materials: [link]

Evidence Base for Conference Approach: Multispecialty case conferences, sometimes called tumor boards in cancer care or multidisciplinary team meetings in other contexts, have been widely implemented in care delivery. For cancer care, previous research indicates that these conferences improve appropriate treatment selection, while the literature on their effectiveness is limited. Multispecialty case conferences, as a component of a comprehensive initiative to improve adult scoliosis surgery outcomes, have been shown to reduce complications. Implementation studies have identified key characteristics to promote conference use, including provider and administrator consensus on usefulness and buy-in to integrating conferences into clinical workflows.

**Refined Nudge Prototype: Preop Check Email for Hypothetical Surgeon / Patient**

***Subject Line:*** Please Review Preop Check for Surgical Patient

***Body of email***

Dear Dr. [Name],

Please review the appropriateness recommendations below for your planned operation. If the recommendations differ from what you might have been considering, please:

- Explore the recommendations further using an **online appropriateness calculator**:
  - Scoliosis Tool: [link]
  - Spondylolisthesis Tool: [link]
  - Supporting materials: [link]
- **Addend your last visit note** with information that might have influenced your procedure selection.
- Join our **bimonthly multispecialty case conference**, where spine surgeons and other experts in spine conditions debate appropriate surgical options for diverse and often challenging clinical situations.
- **Share your feedback:** email our Clinical Implementation Leader, [name], at [email].

Sincerely,

[Site leader name]

**Patient:** [Name] **MRN:** [MRN]

**Diagnosis:** [Scoliosis and/or spondylolisthesis] **OR Date:** [Planned date of surgery]

[Note: Below is an original drawing by the study investigators that depicts the resulting information.]


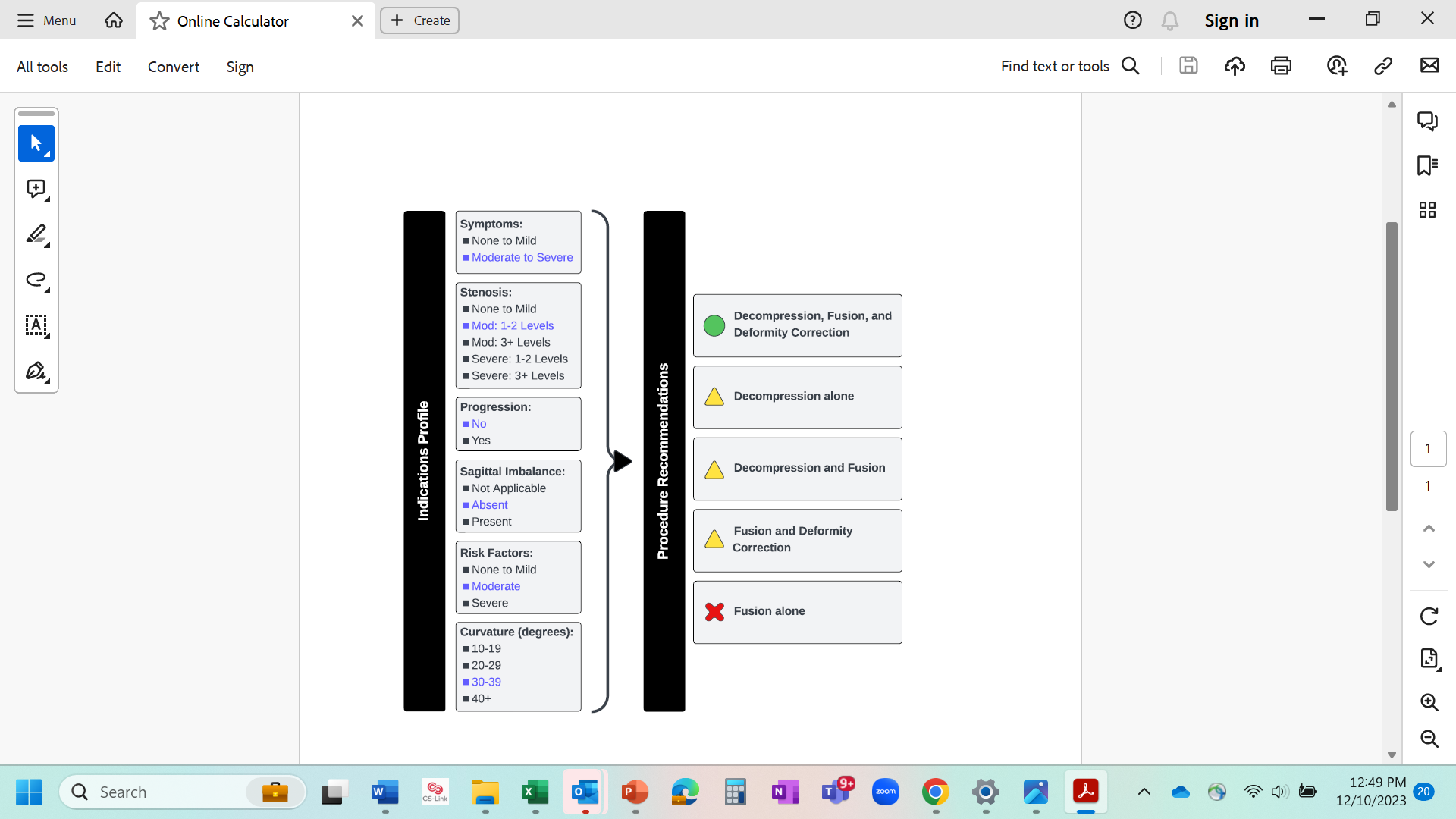

Supplement: S5 File — (DOCX) [file pone.0300475.s005.docx]
