# Supplementary material for: Surgical appropriateness nudges: Developing behavioral science nudges to integrate appropriateness criteria into the decision making of spine surgeons
Source: PLoS One. 2024 Apr 19;19(4):e0300475. doi: 10.1371/journal.pone.0300475 (PMC11029649; doi:10.1371/journal.pone.0300475)
Supplement: S6 File — (DOCX) [file pone.0300475.s006.docx]

**S6 File: Pilot Testing of Refined Nudge Prototypes: Survey Items and Results**

**Mean Responses and Standard Deviation (SD), N=5 Spine Surgeons**

| ***Nudge Prototype and Associated Survey Items* (1-5 scale where 5=strongly agree)** | | ***Mean (SD)*** |
| --- | --- | --- |
| **Preoperative Appropriateness Check** | |  |
| *Acceptability:* | |  |
|  | 1. I would approve of use the preoperative appropriateness check in my practice | 4.0 (0.7) |
|  | 1. I would feel comfortable receiving this preoperative appropriateness check | 4.2 (0.4) |
|  | 1. This preoperative appropriateness check would help me feel more supported in my decision-making | 3.8 (0.8) |
| *Applicability:* | |  |
|  | 1. This preoperative appropriateness check is applicable to my practice | 3.8 (0.4) |
|  | 1. This preoperative appropriateness check would be helpful for me | 3.8 (0.4) |
|  | 1. This preoperative appropriateness check would be helpful for other spine surgeons | 3.8 (0.4) |
| *Feasibility:* | |  |
|  | 1. This preoperative appropriateness check seems like it could be implemented in surgical practice | 3.8 (0.4) |
|  | 1. The mockup personalized preoperative appropriateness checks seemed like they would be easy for me to use | 4.0 (0.0) |
| **Online Appropriateness Calculators** | |  |
| *Acceptability:* | |  |
|  | 1. I would approve of use these appropriateness calculators in my practice | 3.8 (0.4) |
|  | 1. I would feel comfortable receiving recommendations from these appropriateness calculators | 4.0 (0.0) |
|  | 1. These appropriateness calculators would help me feel more supported in my decision-making | 3.8 (0.4) |
| *Applicability:* | |  |
|  | 1. These appropriateness calculators are applicable to my practice | 3.8 (0.4) |
|  | 1. These appropriateness calculators would be helpful for me | 3.8 (0.4) |
|  | 1. These appropriateness calculators would be helpful for other spine surgeons | 3.8 (0.4) |
| *Feasibility:* | |  |
|  | 1. These appropriateness calculators seem like they could be implemented in surgical practice | 3.8 (0.4) |
|  | 1. These appropriateness calculators were easy for me to use | 4.0 (0.0) |
| **Individualized Surgeon Score card** | |  |
| *Acceptability:* | |  |
|  | 1. I would approve of use this score card in my practice | 4.0 (0.0) |
|  | 1. I would feel comfortable receiving this score card | 4.2 (0.4) |
|  | 1. This score card would help me feel more supported in my decision-making | 3.8 (0.4) |
| *Applicability:* | |  |
|  | 1. This score card is applicable to my practice | 3.8 (0.4) |
|  | 1. This score card would be helpful for me | 3.8 (0.4) |
|  | 1. This score card would be helpful for other spine surgeons | 3.8 (0.4) |
| *Feasibility:* | |  |
|  | 1. This score card seems like it could be implemented in surgical practice | 3.8 (0.4) |
|  | 1. The score card was easy for me to use | 3.8 (0.4) |
| **Multispecialty Case Conference** | |  |
| *Acceptability:* | |  |
|  | 1. I would approve of use the case conferences described above in my practice | 4.4 (0.5) |
|  | 1. I would feel comfortable participating in the case conferences described above | 4.4 (0.5) |
|  | 1. The case conferences described above would help me feel more supported in my decision-making | 4.4 (0.5) |
| *Applicability:* | |  |
|  | 1. The case conferences described above is applicable to my practice | 4.4 (0.5) |
|  | 1. The case conferences described above would be helpful for me | 4.4 (0.5) |
|  | 1. The case conferences described above would be helpful for other spine surgeons | 4.2 (0.8) |
| *Feasibility:* | |  |
|  | 1. The case conferences described above seems like it could be implemented in surgical practice | 4.4 (0.7) |
|  | 1. The case conferences described above seemed like they would be easy for me to use | 4.4 (0.5) |
